# Compound Kushen injection reduces severity of radiation-induced gastrointestinal mucositis in rats

**DOI:** 10.3389/fonc.2022.929735

**Published:** 2022-08-11

**Authors:** Yuka Harata-Lee, Zhipeng Qu, Emma Bateman, Xi Xiao, Marianne D. Keller, Joanne Bowen, Wei Wang, David L. Adelson

**Affiliations:** ^1^ School of Biological Sciences, University of Adelaide, Adelaide, SA, Australia; ^2^ School of Biomedicine, University of Adelaide, Adelaide, SA, Australia; ^3^ Preclinical, Imaging and Research Laboratories (PIRL), South Australian Health and Medical Research Institute, Adelaide, SA, Australia; ^4^ Zhendong Research Institute, Zhendong Pharmaceutical, Beijing, China

**Keywords:** Compound Kushen injection, gastrointestinal mucositis, radiation injury, traditional Chinese medicine, inflammatory markers

## Abstract

Mucositis, or damage/injury to mucous membranes of the alimentary, respiratory, or genitourinary tract, is the major side effect associated with anticancer radiotherapies. Because there is no effective treatment for mucositis at present, this is a particular issue as it limits the dose of therapy in cancer patients and significantly affects their quality of life. Gastrointestinal mucositis (GIM) occurs in patients receiving radiotherapies to treat cancers of the stomach, abdomen, and pelvis. It involves inflammation and ulceration of the gastrointestinal (GI) tract causing diarrhea, nausea and vomiting, abdominal pain, and bloating. However, there is currently no effective treatment for this debilitating condition. In this study, we investigated the potential of a type of traditional Chinese medicine (TCM), compound Kushen injection (CKI), as a treatment for GIM. It has previously been shown that major groups of chemical compounds found in CKI have anti-inflammatory effects and are capable of inhibiting the expression of pro-inflammatory cytokines. Intraperitoneal administration of CKI to Sprague Dawley (SD) rats that concurrently received abdominal irradiation over five fractions resulted in reduced severity of GIM symptoms compared to rats administered a vehicle control. Histological examination of the intestinal tissues revealed significantly less damaged villus epithelium in CKI-administered rats that had reduced numbers of apoptotic cells in the crypts. Furthermore, it was also found that CKI treatment led to decreased levels of inflammatory factors including lower levels of interleukin (IL)-1β and IL-6 as well as myeloperoxidase (MPO)-producing cells in the intestinal mucosa. Together, our data indicate a novel effect of CKI to reduce the symptoms of radiation-induced GIM by inhibiting inflammation in the mucosa and apoptosis of epithelial cells.

## 1 Introduction

Radiotherapy often results in serious side effects for cancer patients. GI mucositis (GIM), or damage/injury to mucous membranes of the GI tract, is the major side effect in patients with abdominal or pelvic area tumors and there is no effective treatment for it. This is a significant concern, as it limits the dose of therapy in those patients and has a major effect on their quality of life (1, 2).

While the precise molecular and cellular mechanisms involved in radiation-related mucositis are not fully understood, it is clear that radiation induces cell death in both tumor and healthy tissues. As the GI epithelial cells are highly proliferative, these mucosae are more prone to mucositis. According to the five-stage model of pathogenesis of mucositis (3, 4), radiation induces the generation of reactive oxygen species (ROS), which leads to the initiation stage of mucositis by causing DNA damage and apoptosis of epithelial cells. At the same time, some transcription factors such as NFκB also become activated and upregulated (4, 5). One group of genes targeted by NFκB are pro-inflammatory cytokines. Several studies have shown upregulation of cytokines such as IL-1β, IL-6, and tumor necrosis factor (TNF)α at mRNA and/or protein levels in alimentary tract mucosa of irradiated animals (6–8). Once pro-inflammatory cytokines are upregulated, they have been suggested to provide positive feedback by activating NFκB in other cells leading to further activation of inflammatory pathways (4, 5, 8). Furthermore, other studies also provide evidence that upregulation of these inflammatory cytokines can result in increased recruitment and activation of innate pro-inflammatory leukocytes (9, 10) producing more tissue damaging enzymes (4, 11), leading to amplified tissue injury culminating in the ulcerative phase. The final phase is healing, although residual changes in the mucosa are long-standing and increase the risk of subsequent injury on radiation exposure. Although significant progress in mucositis research using novel animal models has shed some light on the pathobiology of mucositis in the last decade, effective treatments are truly limited (12), and there is an urgent demand for a therapy that can be used to suppress one or more of these pathological pathways.

CKI is a type of TCM and is prepared from the roots of two medicinal herbs, Kushen (*Radix sophorae flavescentis*) and Baituling (*Rhizoma smilacis glabrae*), as an injectable liquid. CKI has been approved for use in China since 1995 by the State Food and Drug Administration of China. Since then, it has been used to treat solid tumors, inflammation, and other diseases and has also been shown to be effective in improving anti-cancer therapies (chemo- or radiotherapy) when used in combination (13–15). Furthermore, major groups of chemical compounds found in Kushen, including alkaloids and flavonoids, have been widely shown by various studies to have anti-inflammatory effects and be capable of inhibiting the expression of pro-inflammatory cytokines (16–20). The central aim of this study is to investigate the effect of CKI on GIM triggered by radiotherapy and to elucidate its mechanisms of action by establishing and using a rat model of radiation-induced GIM.

## 2 Materials and methods

### 2.1 Compound Kushen injection and vehicle control

CKI (Batch No. 20181034) was obtained from Zhendong Pharmaceutical Co. Ltd (Shanxi, China). The roots of *Radix sophorae flavescentis* and *Rhizoma smilacis glabrae* were added to the extraction process at a 7:3 ratio and 1 ml of CKI contains the equivalent of approximately 1.4 g of *Radix sophorae flavescentis* and 0.6 g of *Rhizoma smilacis glabrae*. Vehicle control (VC) was prepared with 0.25% Tween 80 (Sigma Aldrich, MO, USA) and 25 mM HEPES (Thermo Fisher Scientific, MA, USA) in sterile H_2_O.

### 2.2 Rats

Male SD rats were purchased from the Bioresources (South Australian Health and Medical Research Institute (SAHMRI), South Australia (SA), Australia). They were kept in specific pathogen-free conditions.

### 2.3 Induction of GIM in SD rats and CKI administration

All the studies using animals were approved by the Institutional Animal Ethics Committee of SAHMRI (SAM419.19 approved on 9 September 2019 and SAM452.19 approved on 1 August 2020). For irradiation of rats’ abdomens, we designed and constructed a rat holder made from lead with a window that exposes the abdomen while the rest of the body is protected from radiation (**Supplementary Figure 1a**). Male SD rats aged 8 weeks were anesthetized and placed in the lead capsule. Their abdomens were irradiated with five fractions of 4 Gy each (1 fraction per day for 5 consecutive days (day 0 to day 4) to receive a total of 20 Gy. Throughout this study, the “day X post irradiation” means X days after the first fraction of irradiation (day 0). For intervention studies, the rats were also injected intraperitoneally with either CKI (2 ml/kg or 3 ml/kg body weight) or vehicle control (3 ml/kg) from day 0 of irradiation for 7 consecutive days.

### 2.4 Monitoring and scoring of GIM

The irradiated rats were monitored daily for the development of diarrhea and given a score each day as 0 = normal stool, 0.5 = mucous stool, 1 = mild diarrhea with soft stools, 1.5 = mild diarrhea with loose stools and slight staining of perianal fur, 2 = moderate diarrhea with loose stools and perianal staining of fur, 2.5 = moderate diarrhea with loose stools and extended perianal staining of fur, and 3 = severe diarrhea with watery stools and fur staining incorporating hind legs [modified from (21)]. Monitoring of diarrhea was continued until the day of euthanasia.

### 2.5 Tissue preparations for histological analyses

On the indicated days, post-irradiation groups of rats were euthanized and small and large intestines were removed and flushed with cold saline. Approximately 5- to 8-mm pieces were cut from the duodenum, jejunum, ileum, and colon. Tissues were fixed in 10% neutral buffered formalin (Thermo Fisher Scientific) before being sent to Adelaide Histology Services (South Australia, Australia) for tissue processing and paraffin embedding.

### 2.6 Hematoxylin and eosin staining of intestinal sections

Formalin-fixed, paraffin-embedded tissue sections (4 μm thickness) were first dewaxed with histolene and rinsed through a graded ethanol series before being stained in Mayer’s hematoxylin solution (Sigma Aldrich). The nuclear staining was defined in acid alcohol [1% HCl (v/v) in 70% ethanol (w/v)] and blued with Scott’s tap water. The sections were stained with Eosin, dehydrated through a graded ethanol series, and cleared in histolene again before being mounted with DPX (Sigma Aldrich). The resulting slides were scanned with a NanoZoomer Digital Slide Scanner and the images were analyzed on NDP.view2 software (Hamamatsu Photonics, Japan). The scanned images were scored as follows to quantify the extent of radiation-induced damage as “radiation injury score (RIS)”: villous ablation (for small intestine; 0/1) OR fold flattening (for colon; out of 2), crypt ablation (0/1), mucosal ulceration (out of 3), apoptosis (out of 2), epithelial atypia (out of 3), lymphatic congestion (out of 2), and inflammation (out of 3) (22).

### 2.7 Immunohistochemistry for intestinal sections

For immunohistochemical (IHC) analyses, the following primary antibodies were purchased from Abcam (United Kingdom): rabbit anti-Caspase-3 (polyclonal), rabbit anti-Ki-67 (SP6), rabbit anti-MPO (polyclonal), rabbit anti-IL-6 (polyclonal), and rabbit anti-IL-1β (polyclonal); biotinylated goat anti-rabbit IgG (H+L) was purchased from Vector Laboratories (CA, USA). Formalin-fixed, paraffin-embedded tissue sections (4 μm thickness) were first dewaxed with histolene and rinsed through a graded ethanol series. Heat-mediated antigen retrieval was performed in citric acid solution (2.1% w/v, pH 6). Endogenous peroxidase, antibodies, avidin, and biotin were blocked using 3% H_2_O_2_ in methanol (v/v), normal goat serum (Vector Laboratories), and Avidin/Biotin Blocking kit (Vector Laboratories), respectively. Anti-Caspase-3, anti-Ki67, and anti-MPO were applied at 1/100 dilution and anti-IL-6 and anti-IL-1β were applied at 1/800 dilution to the tissue sections. The primary antibodies were detected with biotinylated anti-rabbit IgG at 1/200 dilution and then labeled with ABC peroxidase kit (Vector Laboratories). The signals were visualized using the DAB Substrate System (Sigma-Aldrich). Cell nuclei were counterstained with hematoxylin and the slides were scanned as described above. For MPO staining, the number of MPO-positive cells per mm^2^ was counted and cytokine-stained sections were analyzed for the intensity of staining to provide a score out of 4 (0 = no staining, 1 = weak staining, 2 = moderate staining, 3 = strong staining, and 4 = intense staining). For the analyses of apoptosis levels in the intestinal mucosa, the average numbers of Caspase-3-positive cell per crypt were calculated as (number of positive cell per field of view (FOV))/(number of crypts per FOV). For proliferation levels, average percentages of proliferating cells in a crypt were calculated as the number of Ki67-positive cells/number of cells in a crypt per FOV.

### 2.8 Statistical analyses

All statistical analyses were performed using GraphPad Prism version 9.0.2 for Mac OS X, GraphPad Software, San Diego, California USA, www.graphpad.com. For time course data sets (XY line graphs), two-way ANOVA was used to compare CKI-treated groups to vehicle control groups. Unpaired *t*-test was used for endpoint data sets (column bar graphs).

## 3 Results

### 3.1 Induction of radiation-induced gastrointestinal mucositis in Sprague Dawley rats

**Figure 1:**
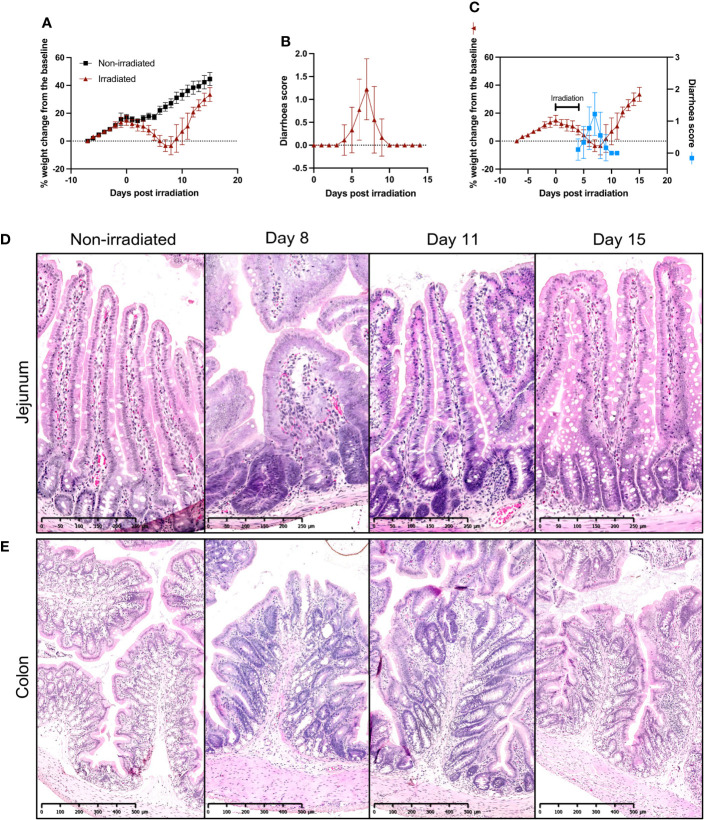
Establishment of fractionated radiation-induced GIM model in SD rats. **(A)** Body weight changes of irradiated and non-irradiated SD rats. Eight-week-old SD rats received five fractions of 4-Gy radiation on their abdomen. The rats were weighed and recorded daily. **(B)** Diarrhea scores of irradiated SD rats. The animals were monitored daily for the presence and degree of diarrhea and given a score according to the scheme. **(C)** Overlay of body weight change and diarrhea scores of irradiated SD rats. **(D, E)** Representative images of H & E-stained jejunum **(D)** and colon **(E)** sections during GIM. The intestines were collected from irradiated rats on days 8, 11, and 15 and embedded in paraffin for H & E staining. Data are presented as mean ± SD.

In order to assess the potential of CKI as a preventative treatment for radiation-induced GIM, an experimental model using SD rats was initially established where the optimal dose of radiation to efficiently induce GIM in SD rats was determined and the disease kinetics were examined. The rats that received five fractions of 4-Gy irradiation showed gradual body weight reduction starting from the second day of irradiation. More significant loss was observed from day 4, and they reached the peak weight loss on day 7 post irradiation (**Figure 1A**). On the other hand, the non-irradiated rats showed a reduced rate of body weight gain during the 5-day irradiation due to the administration of injectable anesthetics; however, the rate of body weight increase recovered immediately after the completion of irradiation (**Figure 1A**). Most of the irradiated animals started showing symptoms of diarrhea from day 5 with soft/mucous-like stools and the symptoms peaked on day 7 with watery stools and peri-anal staining (**Figure 1B**). The overlay of the above sets of data clearly indicates the correlation between the two symptoms of GIM; both weight loss and diarrhea became apparent from day 4/5, reached the peak on day 7, and then started to improve from day 8, and by day 11, rats were recovering from those symptoms (**Figure 1C**).

On days 8, 11, and 15 post irradiation, rats were euthanized and small sections of duodenum, jejunum, ileum, and colon were collected for histological examinations. Hematoxylin and eosin (H & E) staining of small intestine sections from non-irradiated rats showed healthy intestinal lining with finger-like villi interspersed with crypts in a well-organized pattern and the epithelial layer had goblet cells evenly distributed along the length of the crypts and villi (**Figure 1D** and **Supplementary Figures 1b, c**). However, by day 8 post irradiation, extensive damage was prevalent along the small intestine with blunting and fusion of villi and crypt ablation (**Figure 1D** and **Supplementary Figures 1b, c**). By day 11, epithelial damage was mostly repaired, although in some parts, a slight distortion of villi and crypt structures was still observed. By day 15, mucosal injuries were completely healed (**Figure 1D** and **Supplementary Figures 1b, c**).

In the colon, similar pathological kinetics were observed, where, on day 8, the colon mucosa showed severe damage with shortened colonic folds, swelling of submucosa, crypt ablation, and loss of goblet cells while non-irradiated colon had long colonic folds protruding to the lumen and well-organized crypts and goblet cells (**Figure 1E**). On day 11, although the flattening of the colonic folds was still observable, swelling of submucosa had lessened and more intact goblet cells were found, and by day 15, the colon mucosa displayed similar morphology to that of non-irradiated rats (**Figure 1E**).

### 3.2 Administration of CKI reduces radiation-induced damage in intestinal mucosa

**Figure 2:**
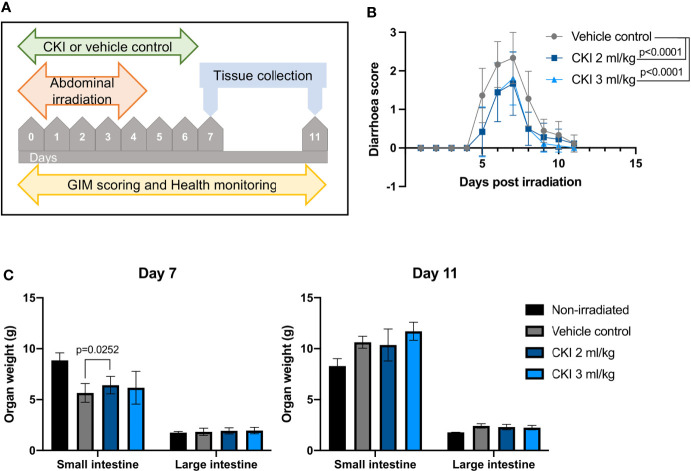
Administration of CKI reduces the severity of radiation-induced GIM. **(A)** Schematic representation of the experimental timeline. **(B)** Diarrhea scores of irradiated SD rats. Rats received five fractions of radiation on their abdomen and were concurrently treated with CKI or vehicle control. **(C)** Weight of the intestines of irradiated rats on day 7 (left) and day 11 (right). Rats were euthanized on day 7 and day 11 post initial irradiation and small and large intestines were collected and weighed. Data are presented as mean ± SD.

Using the established model of radiation-induced GIM in SD rats, we investigated whether CKI administration had any beneficial effect on the symptoms of radiation-induced GIM. SD rats received five fractions of irradiation at 4 Gy per day on their abdomen and were also injected with CKI or VC concurrently with irradiation (**Figure 2A**). The data from monitoring the degree of diarrhea clearly revealed that those rats administered with either 2 ml/kg or 3 ml/kg body weight of CKI developed significantly milder symptoms compared with those that received vehicle control (**Figure 2B**). On day 7, the weight of the small intestine from the rats treated with 2 ml/kg of CKI was significantly higher compared with vehicle control, while the other dose for small intestine and the weight of large intestine did not show any significant differences (**Figure 2C**). This difference in weight of the small intestine may indicate that CKI-treated tissues sustained radiation injury to a lesser degree.

**Figure 3:**
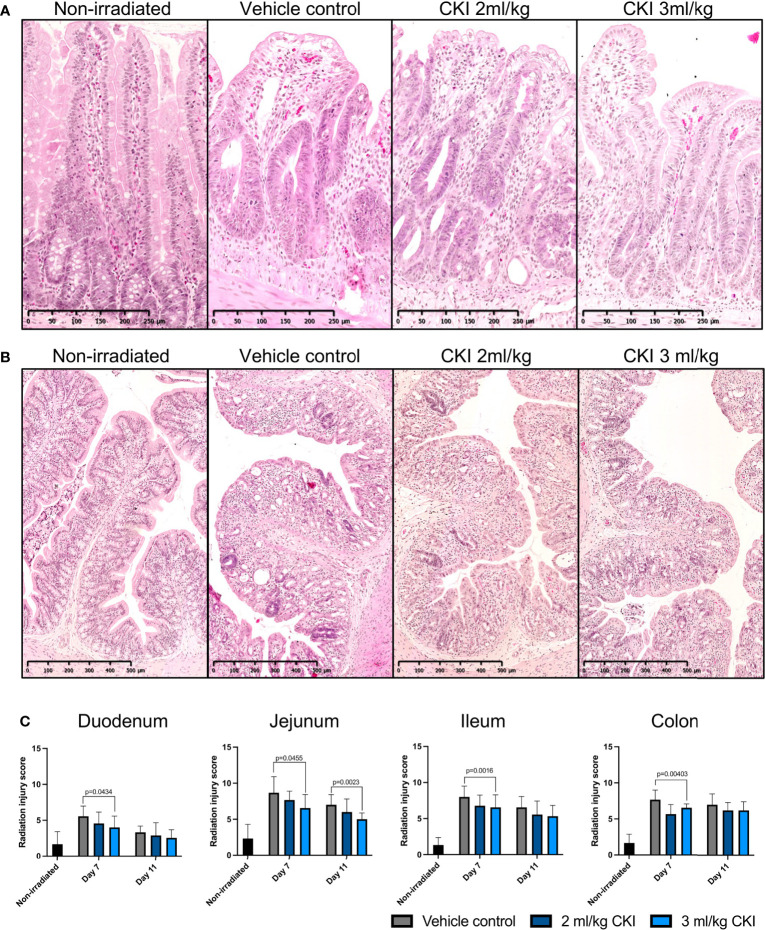
Administration of CKI reduces radiation-induced mucosal damage. **(A, B)** Representative images of H & E-stained jejunum **(A)** and colon **(B)** sections from day 7. The intestines were collected from irradiated rats and paraffin-embedded tissues were sectioned and stained with H & E. **(C)** Radiation injury scores of irradiated intestinal mucosa. The stained sections were analyzed and scored for grade of injury and inflammation as described in *Materials and Methods*. Data are presented as mean ± SD.

On day 7 and day 11 post irradiation, a small section of duodenum, jejunum, ileum, and colon was collected for histological analyses to assess the extent of radiation-induced mucosal injury with or without CKI treatment. H & E staining of the intestinal tissues revealed substantial mucosal damage in all parts of the small intestine with villi blunting and fusion, crypt ablation, and loss of goblet cells in vehicle control-treated rats on day 7, whereas treatment with CKI reduced the damage with greater integrity of villi/crypt structures and more intact goblet cells (**Figure 3A** and **Supplementary Figure 2a**). Similarly in the colon, irradiated mucosa without CKI treatment displayed a high degree of mucosal damage with shortened colonic folds, swelling of submucosa, and severe crypt ablation with loss of goblet cells compared with CKI-treated colon mucosa, where these injuries were less prominent (**Figure 3B**). By day 11 post irradiation, most of the mucosal damage in the intestines had been repaired. In the small intestine treated with vehicle control, some injuries were still observed, whereas those treated with CKI showed villi and crypt structures that were similar to non-irradiated controls (**Supplementary Figure 2b**). The degree of radiation-induced mucosal damage was also quantified and scored as described in *Materials and Methods*. RIS were significantly lower for the rats treated with 3 ml/kg dose of CKI compared with vehicle control in all sections of intestine on day 7 post irradiation (**Figure 3C**). By day 11 post irradiation, no significant differences were observed between vehicle control and CKI-treated groups along the intestine except for in the jejunum where 3 ml/kg CKI treatment still resulted in significantly reduced RIS (**Figure 3C**). Together, these data indicate that administration of CKI to SD rats receiving abdominal irradiation can reduce GIM symptoms by mitigating the mucosal damage of the irradiated intestinal tissues.

### 3.3 CKI protects intestinal mucosa by inhibiting radiation-induced apoptosis and inflammation

**Figure 4:**
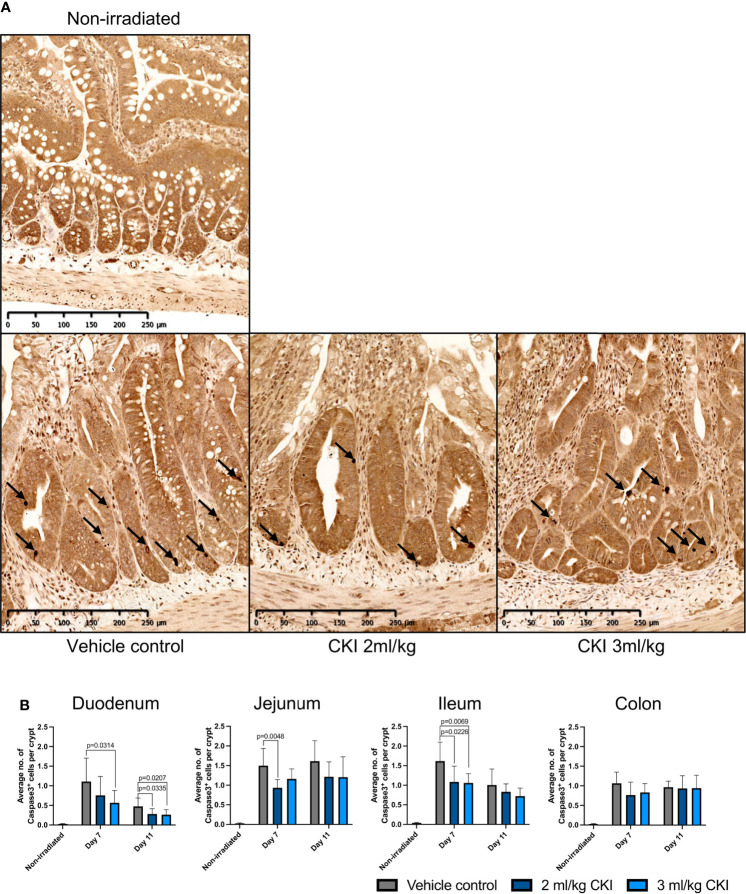
Administration of CKI prevents cell apoptosis in irradiated epithelia. **(A)** Representative IHC images of jejunum sections stained for Caspase-3. The jejunum sections were collected from irradiated rats on day 7 post initial irradiation and paraffin-embedded tissues were sectioned for IHC detection of Caspase-3. **(B)** Average number of Caspase-3-positive cells per crypt. The stained sections of duodenum, jejunum, ileum, and colon were analyzed and the number of Caspase-3-positive cells and crypts was counted in each field of view to calculate the average number of Caspase-3-positive cells per crypt. Data are shown as mean ± SD.

To investigate how administration of CKI resulted in reduced mucosal injuries caused by radiation, the effects of CKI on intestinal epithelial cell apoptosis, proliferation, and mucosal inflammation were examined. Firstly, to examine the effect of CKI on the turnover of epithelial cells in irradiated mucosa, IHC analyses for Caspase-3 and Ki-67 were performed. Caspase-3 staining on irradiated intestinal sections revealed significantly reduced numbers of Caspase-3^+^ cells in the crypts of small intestines that were treated with 2 and/or 3 ml/kg doses of CKI compared with vehicle control on day 7 post irradiation (**Figures 4A, B**, **Supplementary Figures 3a–c**). However, in the colon, there was no difference in the number of Caspase-3^+^ cells in the crypts of CKI-treated and vehicle control-treated tissue (**Figure 4B** and **Supplementary Figure 3d**). In contrast to the levels of apoptosis, cell proliferation marker Ki-67 staining of intestinal sections showed no significant differences in the number of Ki-67^+^ cells in the crypts of small and large intestines between CKI-treated and vehicle control-treated rats (**Supplementary Figure 4**).

**Figure 5:**
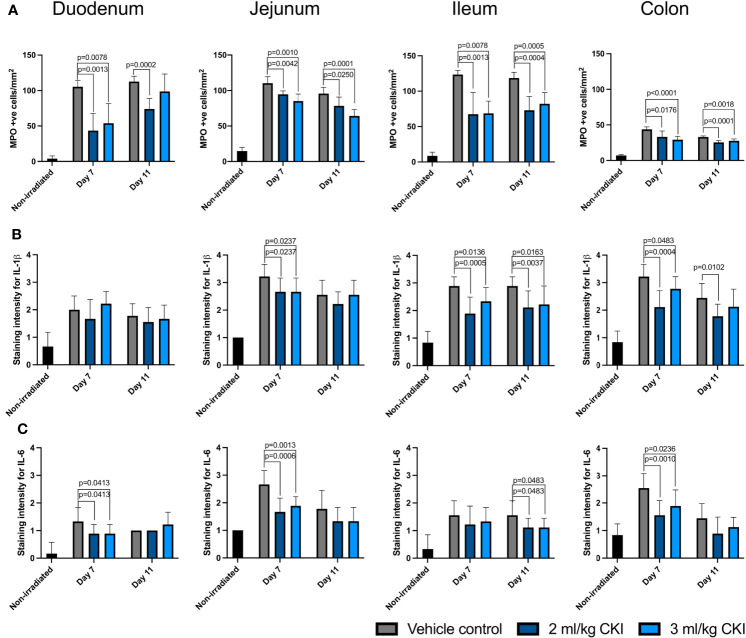
CKI administration reduces the levels of inflammatory factors in irradiated mucosa. **(A)** Number of MPO-positive cells in the irradiated intestine. **(B, C)** Levels of inflammatory cytokines IL-1β **(B)** and IL-6 **(C)** in the irradiated intestine. The intestines were collected from irradiated rats with or without CKI treatment on day 7 and 11 post irradiation and paraffin-embedded tissues were sectioned for IHC detection of inflammatory factors. Data are presented as mean ± SD.

Given that anti-inflammatory effects have been reported for CKI, and tissue injury-induced inflammation is one of the hallmarks of the pathobiology of mucositis, the effect of CKI on the extent of radiation-induced epithelial inflammation was also assessed by IHC detection of the tissue damaging proinflammatory enzyme, MPO, and proinflammatory cytokines, IL-1β and IL-6. The number of MPO^+^ cells was significantly reduced in the irradiated small and large intestinal mucosae with CKI treatment compared those treated with vehicle control (**Figure 5A**, **Supplementary Figures 5a–d**). Furthermore, the levels of IL-1β in CKI-treated animals were significantly lower in jejunum, ileum, and colon, but not in duodenum, on day 7 post irradiation compared with vehicle control-treated rats (**Figure 5B** and **Supplementary Figures 5e–h**). Similarly, the levels of IL-6 in CKI-treated rats were significantly reduced in duodenum, jejunum, and colon, but not in ileum, at the peak of GIM (**Figure 5C** and **Supplementary Figures 5i–l**). Together, these data demonstrate that CKI reduces radiation-induced mucosal inflammation in addition to inhibiting epithelial cell apoptosis, while having no effect on the level of cell proliferation, thus reducing the severity of radiation-induced GIM.

## 4 Discussion

The results of this study indicate a new potential use for CKI as a preventative treatment for GIM resulting from cancer radiotherapy. Our data clearly indicate that when CKI is administered concurrently with fractionated abdominal irradiation, it leads to less severe diarrhea in SD rats by reducing radiation-induced mucosal damage in the intestines. Histological analyses also revealed that CKI acts by inhibiting epithelial cell apoptosis and tissue damage-induced inflammation.

To the best of our knowledge, this is the first study that demonstrates the effectiveness of CKI on GIM in an animal model, and it sheds light on possible mechanisms underlying its effect. Our observations are consistent with a recent investigation by Zhen et al. that indicated that CKI protects skin from radiation-induced injury in patients with nasopharyngeal cancer (23). In addition, two studies that performed meta-analyses on previous randomized controlled clinical trials for efficacy of CKI in lung cancer (24) or esophageal cancer patients (25) receiving radiotherapy also suggest that CKI reduces radiation pneumonitis and esophagitis in those patients.

In this study, our histological analyses showed that CKI inhibits apoptosis in irradiated GI epithelium. However, CKI has been extensively shown to promote apoptosis in our previous studies (26–28) as well as in studies by others (15, 29, 30) in tumor cells. We speculate that this discrepancy results from the fundamental differences between tumor cells and normal epithelial cells in response to various cellular signals including those responsible for cell death resistance. Indeed, Zheng et al. in the aforementioned study performed *in vitro* studies using the non-small cell lung cancer cell line, H1299, and normal human skin fibroblast (HSF) cells that showed that CKI promotes radiation-induced apoptosis in H1299 cells but reverses the induction of apoptosis in normal HSF cells (23). Their data also indicated that CKI exerts this effect by upregulating the pro-apoptotic protein Bim only in tumor cells but not in HSF cells (23). While the mechanisms of differential response to CKI by tumor and non-tumor cells is not fully understood, it is a promising indication that CKI can exert differential effects on cancerous cells and normal epithelium.

In addition, our data also indicated that CKI suppresses GI epithelium damage by reducing radiation-induced inflammation. Kushen has a long history of being used to treat inflammation related conditions and there is growing scientific evidence from various *in vitro* and *in vivo* models that show that Kushen or compounds found in Kushen extract have anti-inflammatory effects (16–20). However, there are limited studies showing the effect of CKI on inflammatory conditions *in vivo*. In the study performed by Zhou et al., CKI was shown to reduce the levels of IL-6 and TNFα in the serum of rats where gastric cancer was induced with N-methyl-N’-nitro-N-nitrosoguanidine (MNNG) (29). In another study by Zhao et al., CKI was also shown to reduce several inflammatory cytokines including IL-6 in sarcoma-bearing mice (31). Our current study provides further evidence and verified the anti-inflammatory effect of CKI *in vivo*.

As CKI is a complex mixture produced from two distinct plants in which more than 20 compounds are found, it is not surprising that CKI has an impact on multiple cellular functions in this GIM model. With such complex mixtures of natural products, molecular interactions between multiple compounds can alter a multitude of biological networks. This has been demonstrated in our previous studies (28, 32). These interactions may also contribute to the differential effects exerted by CKI on normal cells and cancerous cells. To fully understand the mechanisms of action of CKI leading to reduction of symptoms of radiation-induced GIM, it is essential that we gain a better understanding of the effects of CKI on molecular networks and cellular pathways. Nevertheless, our data indicate that CKI has a significant role not only in reducing radiation-induced inflammation but also in reducing tissue damage by reversing radiation-induced cell apoptosis, supporting the view that a better understanding of CKI’s mechanism of action may lead to a better understanding of the pathophysiology of GIM and other conditions involving tissue damage.

## Data availability statement

The original contributions presented in the study are included in the article/**Supplementary Material**. Further inquiries can be directed to the corresponding author.

## Ethics statement

The animal study was reviewed and approved by Institutional Animal Ethics Committee of South Australian Health and Medical Research Institute.

## Author contributions

Conceptualization, YH-L and DA; methodology, YH-L, MK, and JB; formal analysis, YH-L and EB; investigation, YH-L, ZQ, EB, and XX; resources, WW; data curation, YH-L; writing—original draft preparation, YH-L; writing—review and editing, ZQ, JB, and DA; supervision, DA; project administration, DA; funding acquisition, DA. All authors have read and agreed to the published version of the manuscript.

## Funding

This research was funded by Shanxi Zhendong Pharmaceutical Co. Ltd. (UA182520).

## Acknowledgments

The authors would like to thank the staff of Bioresources at South Australian Health and Medical Research Institute for the technical and animal husbandry support.

## Conflict of interest

WW is an employee of Shanxi Zhendong Pharmaceutical Co. Ltd.

The remaining authors declare that the research was conducted in the absence of any commercial or financial relationships that could be construed as a potential conflict of interest.

The funder had no role in the design of the study; in the collection, analyses, or interpretation of data; in the writing of the manuscript; or in the decision to publish the results.

## Publisher’s note

All claims expressed in this article are solely those of the authors and do not necessarily represent those of their affiliated organizations, or those of the publisher, the editors and the reviewers. Any product that may be evaluated in this article, or claim that may be made by its manufacturer, is not guaranteed or endorsed by the publisher.

## Supplementary material

The naming of the files is as followed:

image 1 - SuppFig 1

image 2 - SuppFig 2

image 3 - SuppFig 3

image 4 - SuppFig 4

image 5 - SuppFig 5a-g

image 6 - SuppFig 5h-l.
