# Supplementary material for: Compound Kushen injection reduces severity of radiation-induced gastrointestinal mucositis in rats
Source: Front Oncol. 2022 Aug 11;12:929735. doi: 10.3389/fonc.2022.929735 (PMC9403047; doi:10.3389/fonc.2022.929735)
Supplement: Supplementary file 1 [file Image_1.pdf]

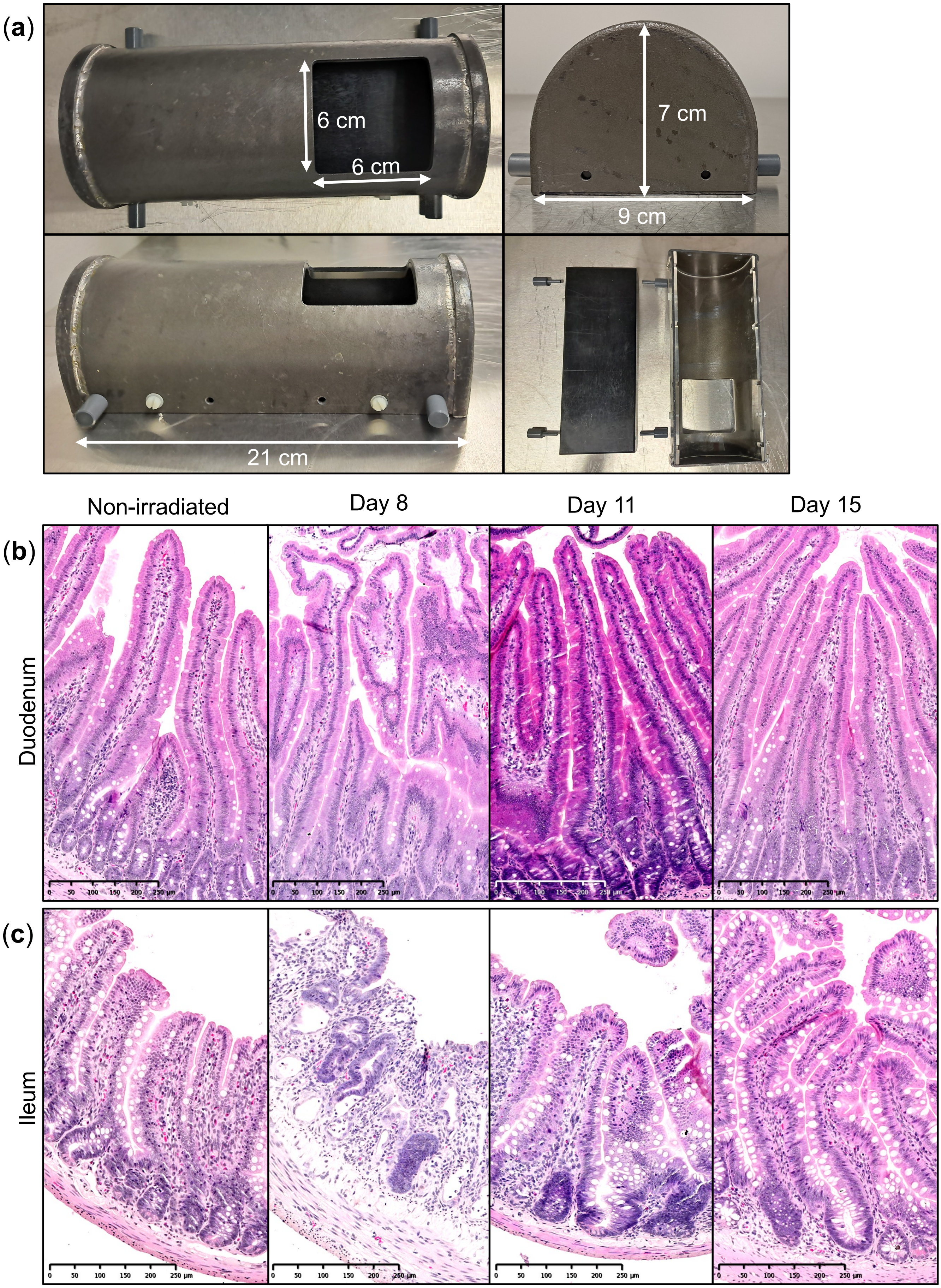

Supplementary figure 1: Establishment of fractionated radiation induced GIM model in SD rats. (**a**) Images of the lead capsule with dimensions. (**b** and **c**) Representative images of H and E stained duodenum (**b**) and ileum (**c**) sections during GIM. The intestines were collected from irradiated rats on days 8, 11 and 15 and embedded in paraffin for H and E staining.
