# Supplementary material for: Compound Kushen injection reduces severity of radiation-induced gastrointestinal mucositis in rats
Source: Front Oncol. 2022 Aug 11;12:929735. doi: 10.3389/fonc.2022.929735 (PMC9403047; doi:10.3389/fonc.2022.929735)
Supplement: Supplementary file 2 [file Image_2.pdf]

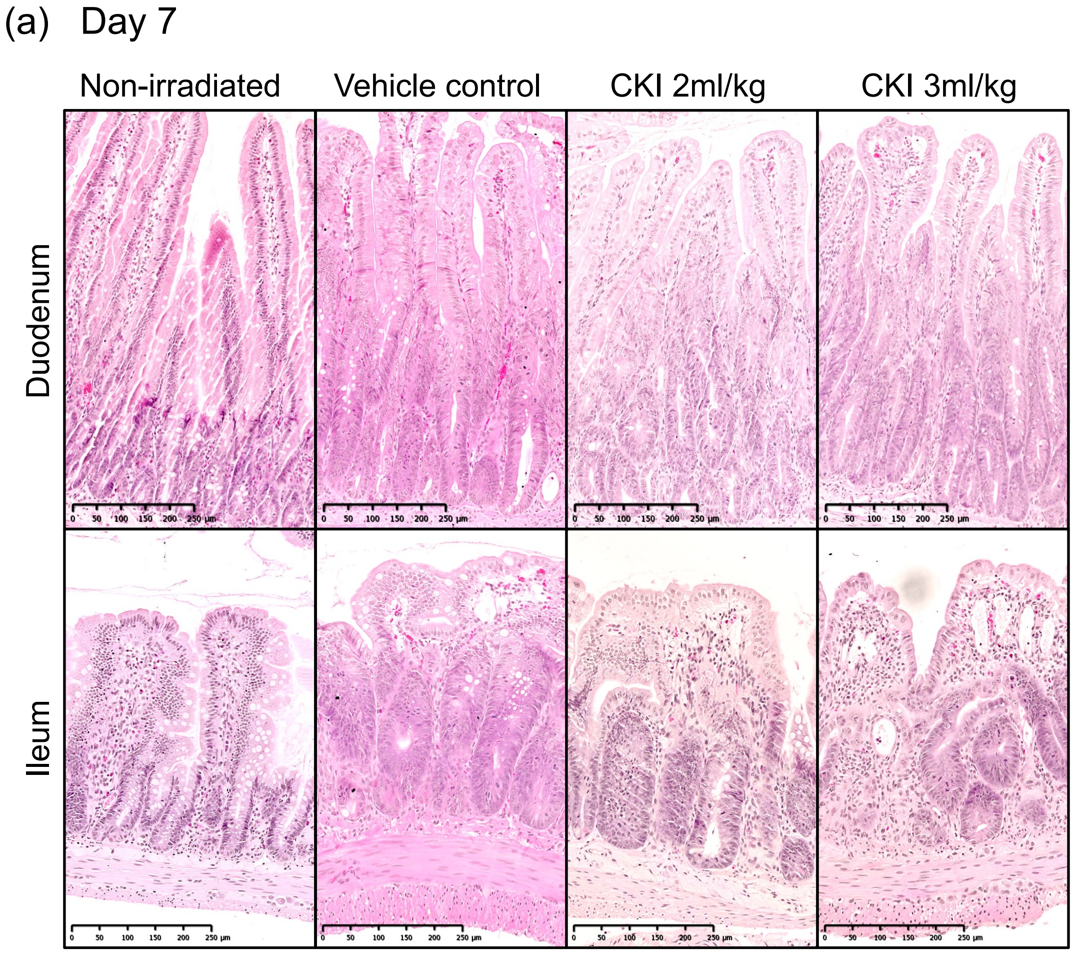
(a) Day 7
Non-irradiated
Vehicle control
CKI 2ml/kg
CKI 3ml/kg
Duodenum
Ileum
0 50 100 150 200 250 µm

(b) Day 11

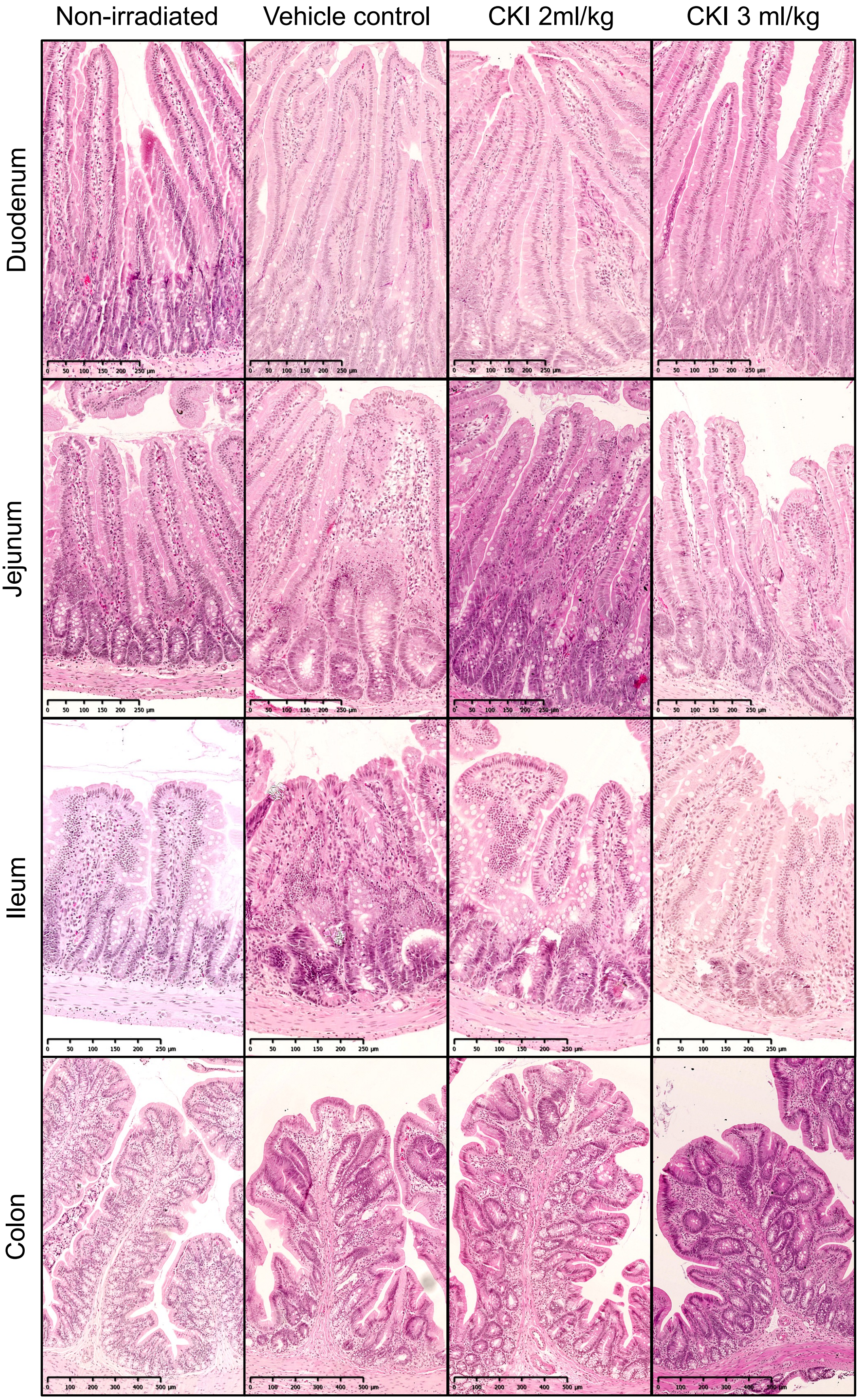

Supplementary figure 2: Administration of CKI reduces radiation induced mucosal damage. Representatives images of H and E stained duodenum and ileum sections from day 7 (**a**) and duodenum, jejunum, ileum and colon sections from day 11 (**b**). The intestines were collected from irradiated rats and paraffin-embedded tissues were sectioned for H & E staining.
