# Supplementary material for: Compound Kushen injection reduces severity of radiation-induced gastrointestinal mucositis in rats
Source: Front Oncol. 2022 Aug 11;12:929735. doi: 10.3389/fonc.2022.929735 (PMC9403047; doi:10.3389/fonc.2022.929735)
Supplement: Supplementary file 3 [file Image_3.pdf]

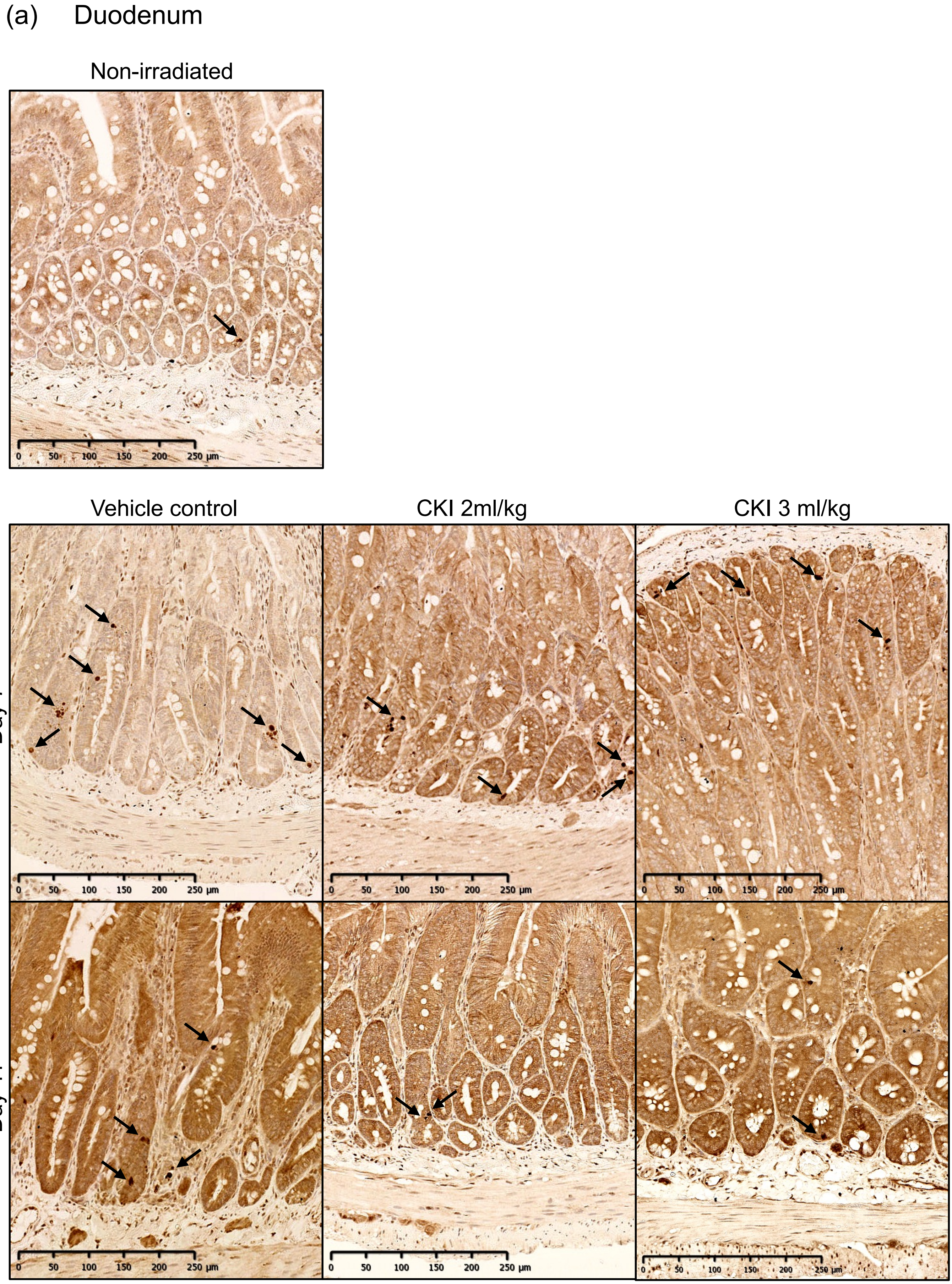
(a) Duodenum
Non-irradiated
Vehicle control
CKI 2ml/kg
CKI 3 ml/kg
Day 7
Day 11
0 50 100 150 200 250 µm

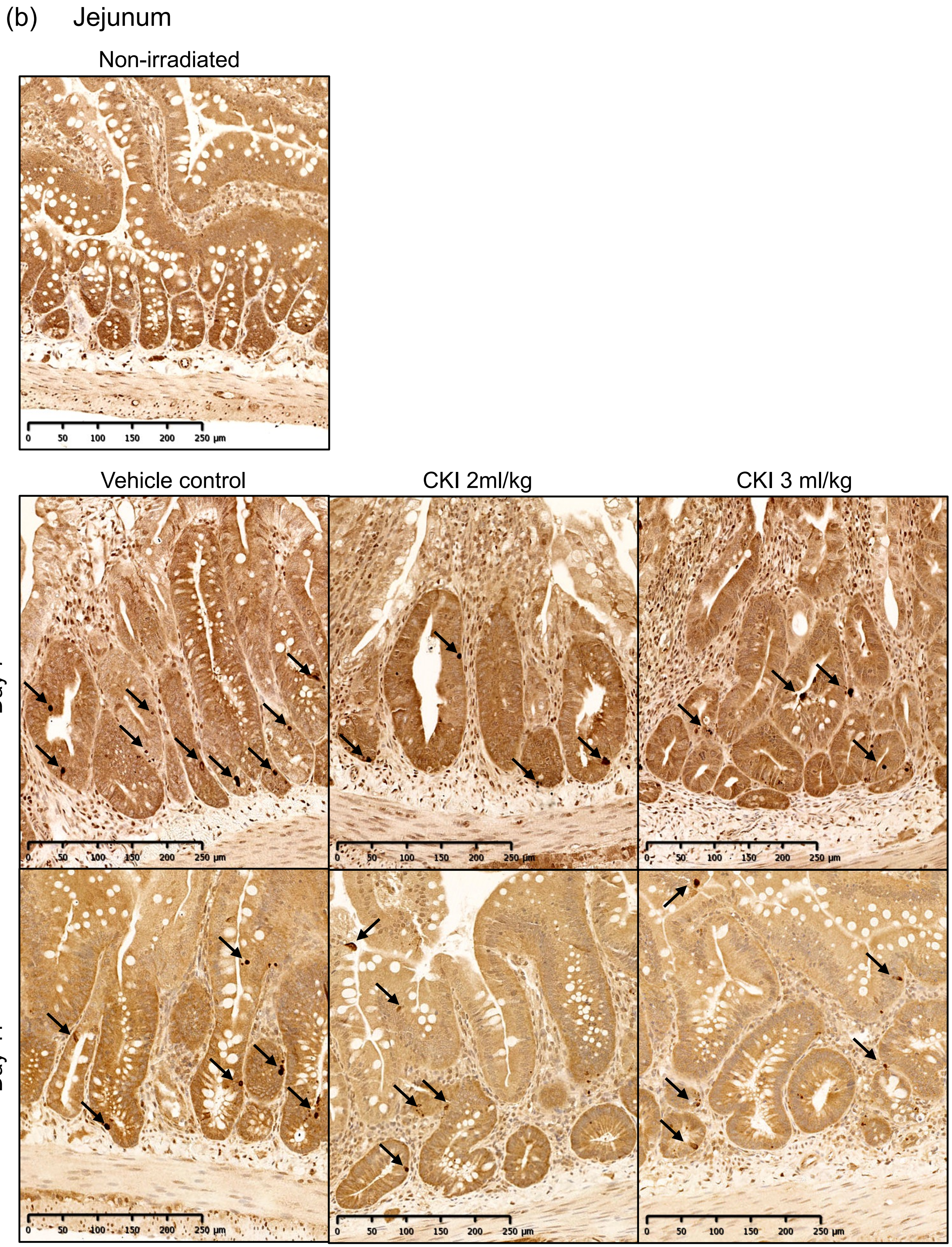
(b) Jejunum
Non-irradiated
0 50 100 150 200 250 µm
Vehicle control
CKI 2ml/kg
CKI 3 ml/kg
Day 7
Day 11

(c) Ileum

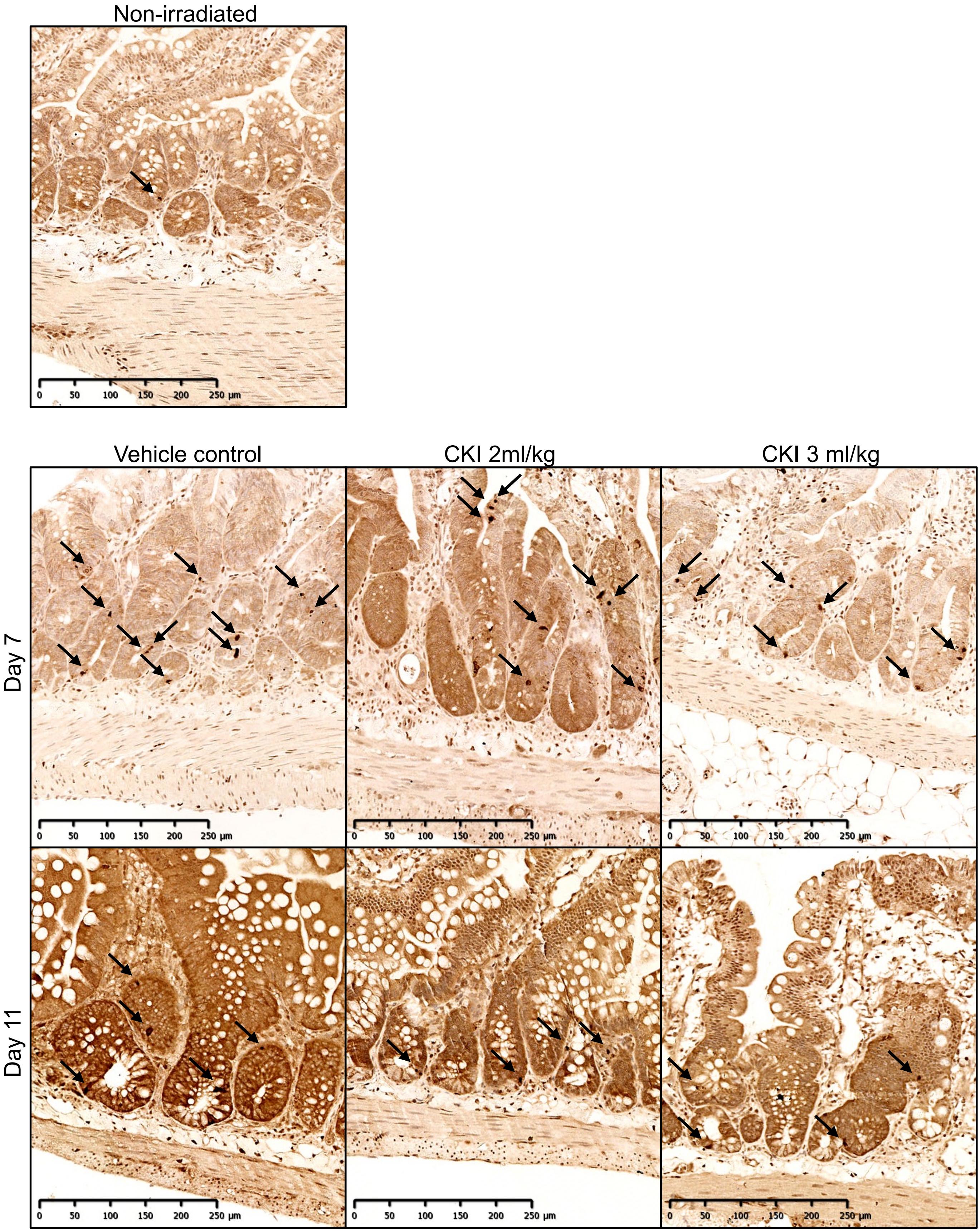
Non-irradiated
Vehicle control
CKI 2ml/kg
CKI 3 ml/kg
Day 7
Day 11
0 50 100 150 200 250 µm

(d) Colon

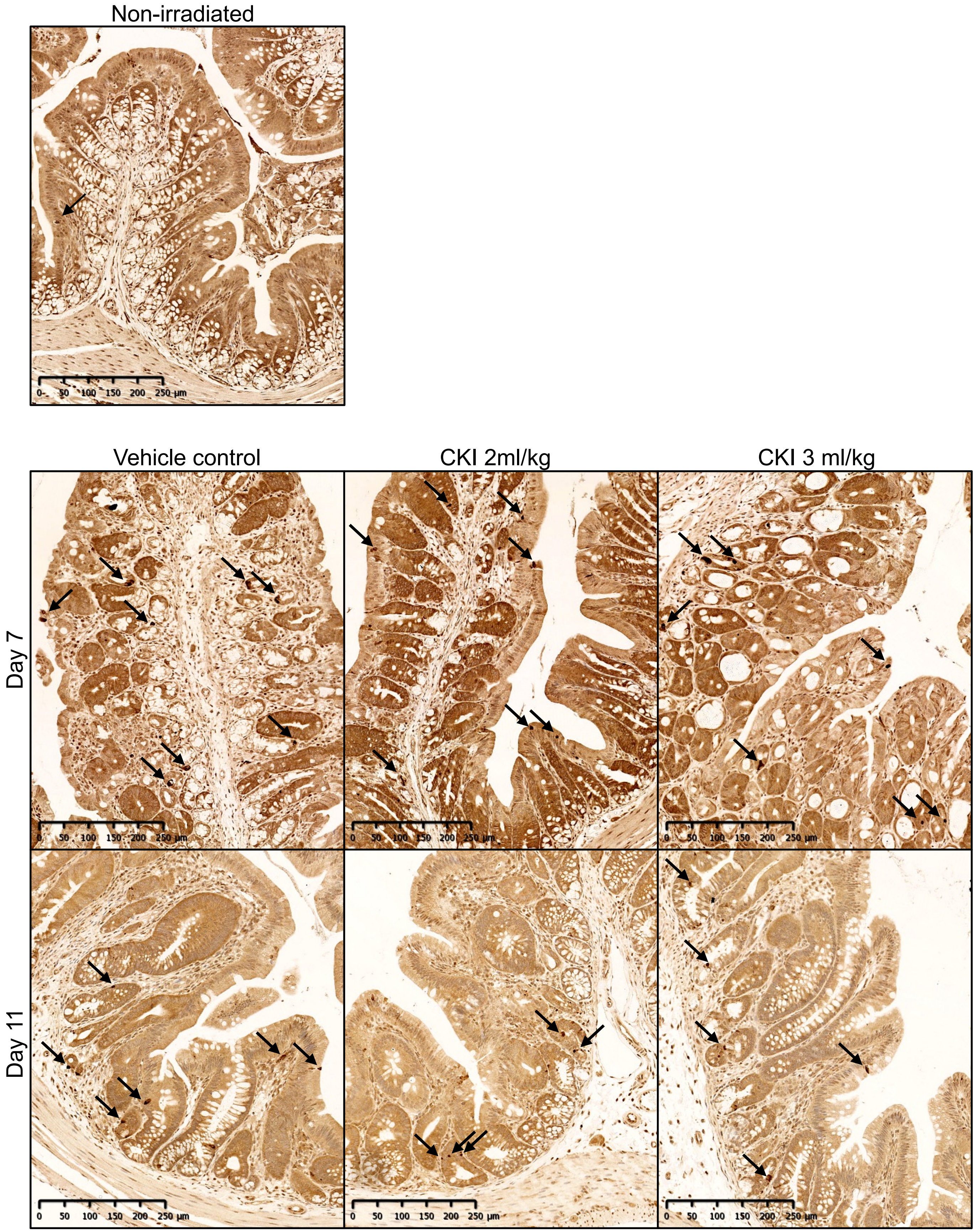

Non-irradiated
0 50 100 150 200 250 µm
Vehicle control
CKI 2ml/kg
CKI 3 ml/kg
Day 7
Day 11

Supplementary figure 3: Administration of CKI prevents cell apoptosis of irradiated epithelia on day 7 post irradiation. Representative IHC images of duodenum (a), jejunum (b), ileum (c) and colon (d) sections stained for Caspase-3. The intestines were collected from irradiated rats on day 7 and 11 post initial irradiation and paraffin-embedded tissues were sectioned for IHC detecting Caspase-3. Note: The images of jejunum for "Non-irradiated" and "Day 7" are presented in the article.
