# Supplementary material for: Compound Kushen injection reduces severity of radiation-induced gastrointestinal mucositis in rats
Source: Front Oncol. 2022 Aug 11;12:929735. doi: 10.3389/fonc.2022.929735 (PMC9403047; doi:10.3389/fonc.2022.929735)
Supplement: Supplementary file 4 [file Image_4.pdf]

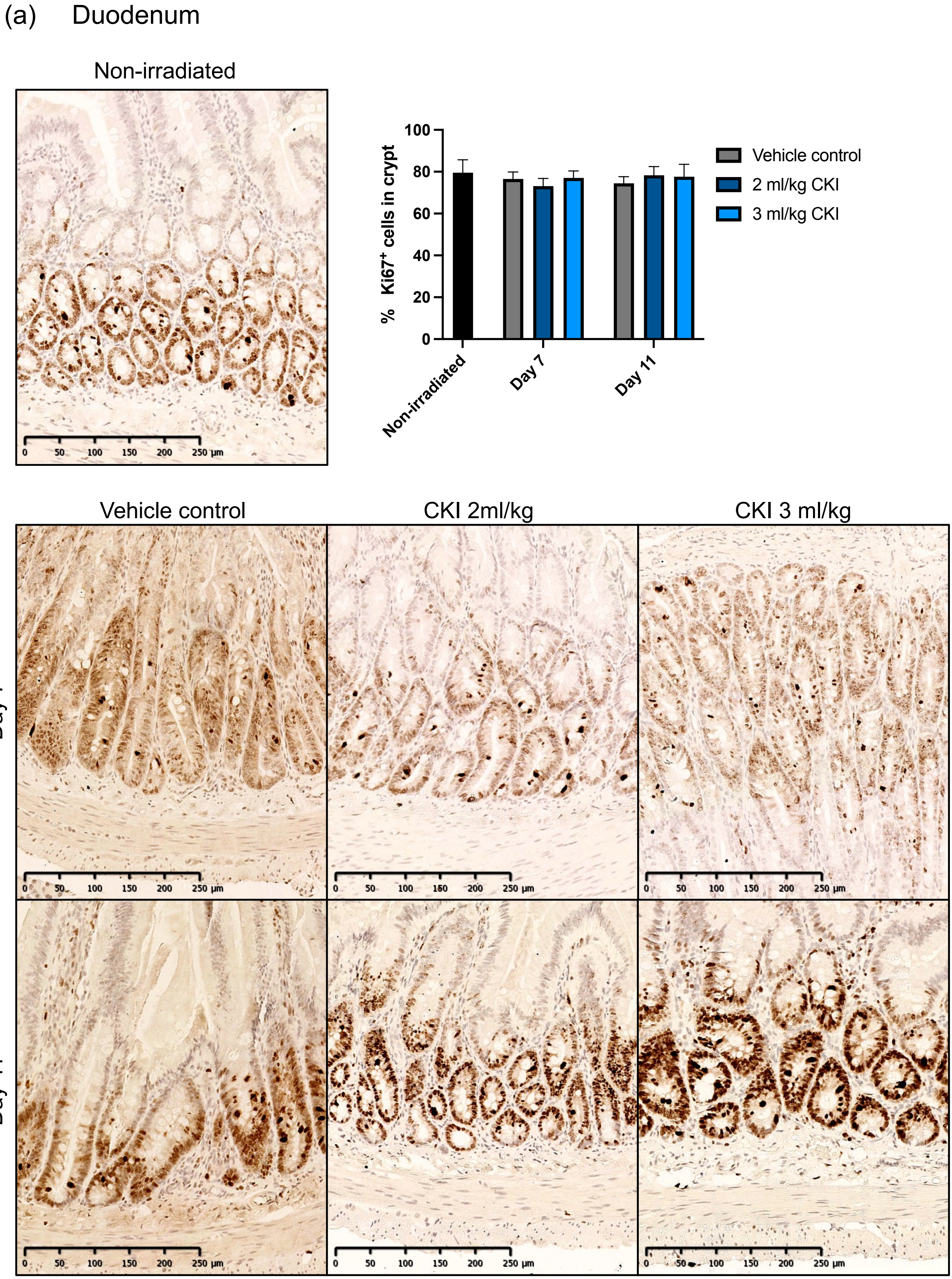
(a) Duodenum
Non-irradiated
% Ki67+ cells in crypt
100
80
60
40
20
0
Non-irradiated
Day 7
Day 11
Vehicle control
2 ml/kg CKI
3 ml/kg CKI
Vehicle control
CKI 2ml/kg
CKI 3 ml/kg
Day 7
Day 11
0 50 100 150 200 250 µm

(b) Jejunum

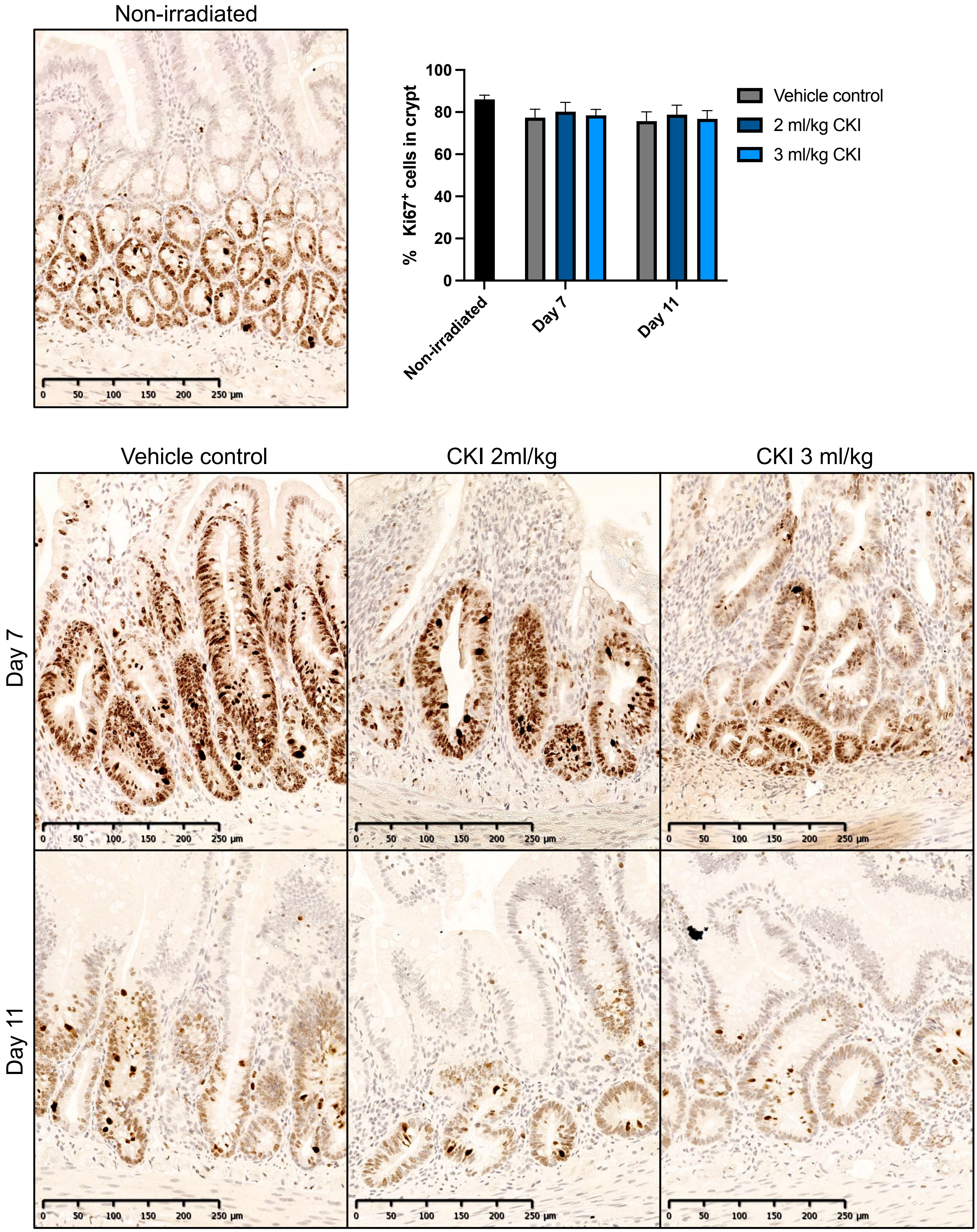

Non-irradiated
0 50 100 150 200 250 µm
% Ki67+ cells in crypt
100
80
60
40
20
0
Non-irradiated
Day 7
Day 11
Vehicle control
2 ml/kg CKI
3 ml/kg CKI
Vehicle control
CKI 2ml/kg
CKI 3 ml/kg
Day 7
Day 11

(c) Ileum

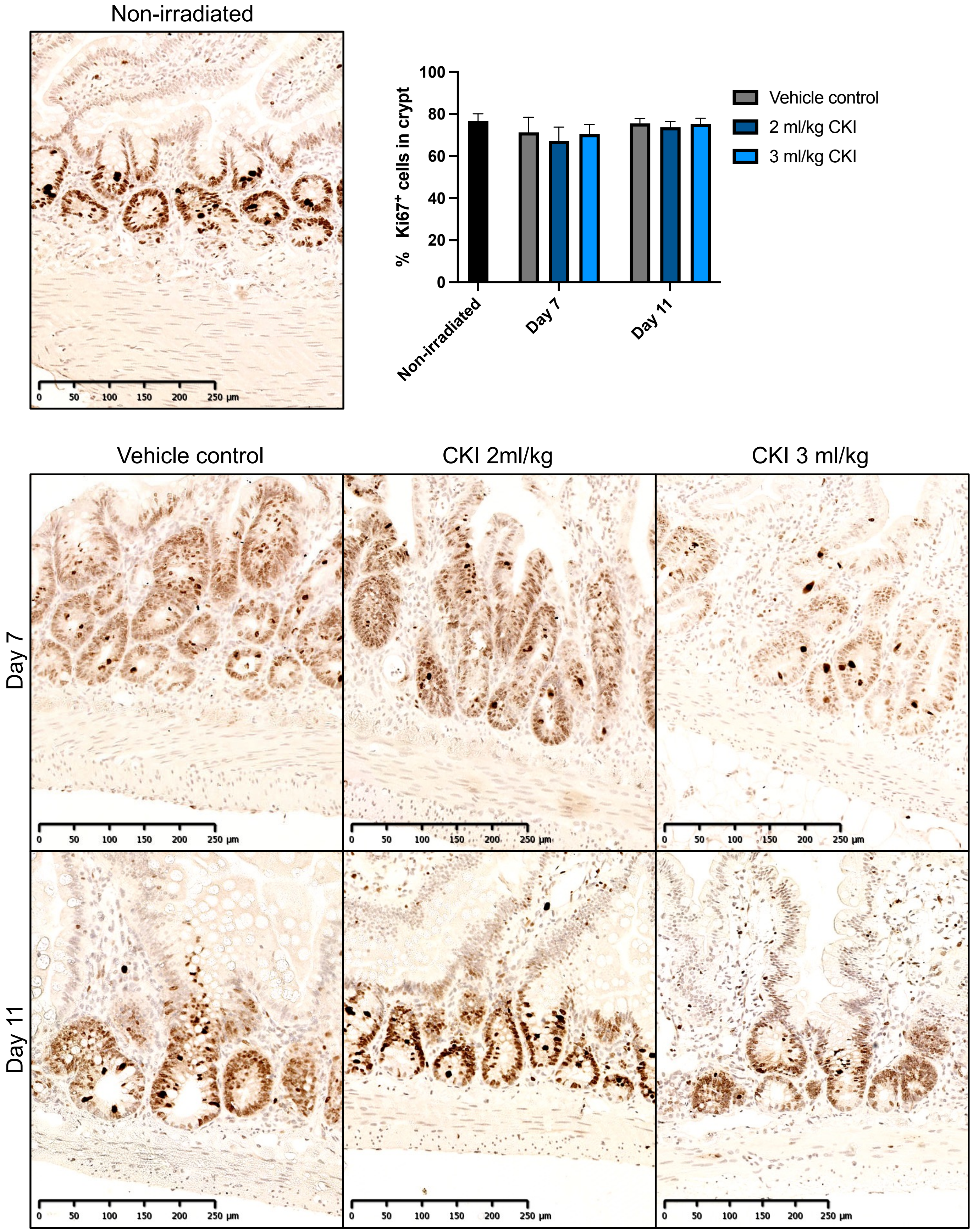
Non-irradiated
0 50 100 150 200 250 µm
% Ki67+ cells in crypt
100
80
60
40
20
0
Non-irradiated
Day 7
Day 11
Vehicle control
2 ml/kg CKI
3 ml/kg CKI
Vehicle control
CKI 2ml/kg
CKI 3 ml/kg
Day 7
Day 11

(d) Colon

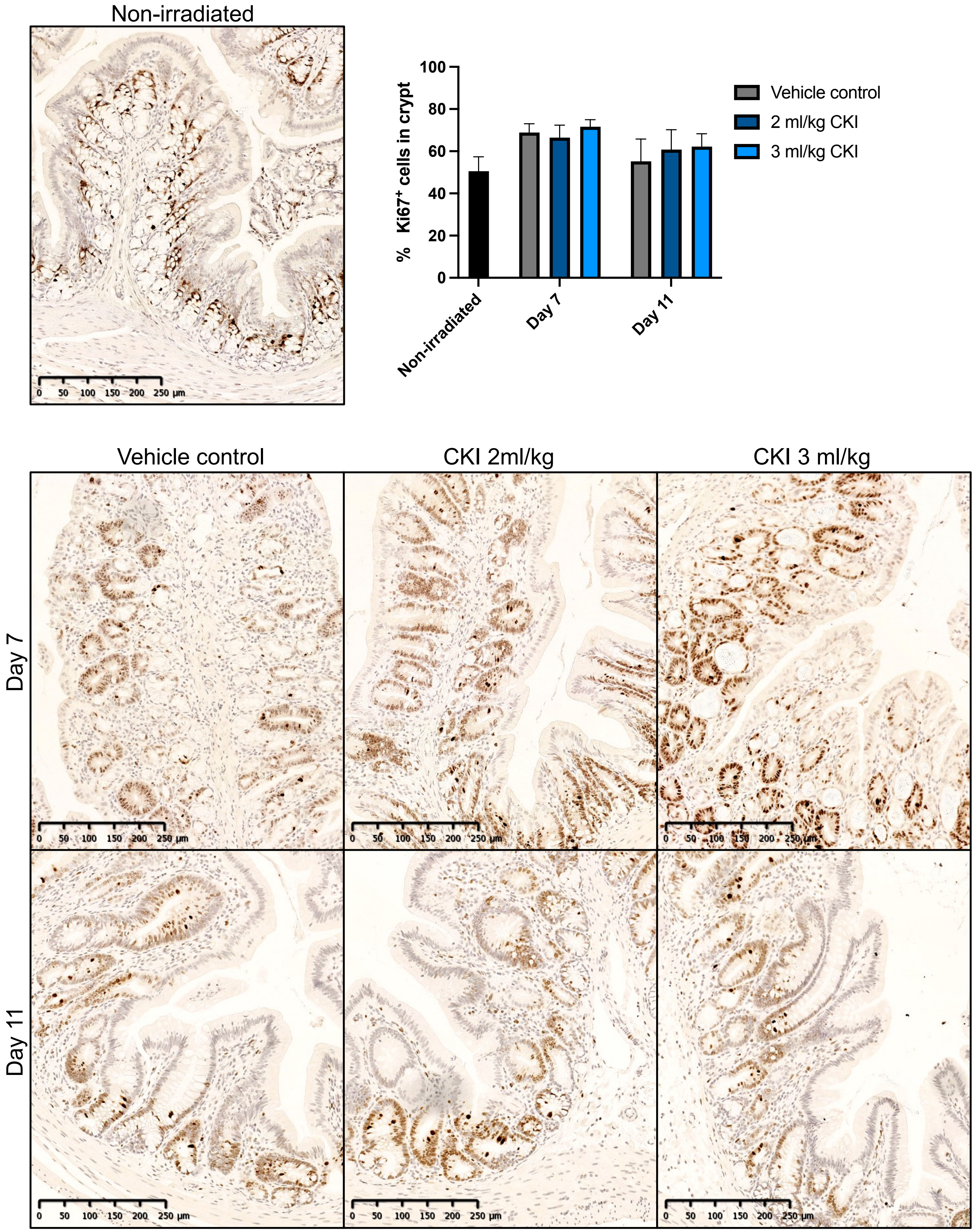

Non-irradiated
0 50 100 150 200 250 µm
100
80
60
40
20
0
% Ki67⁺ cells in crypt
Non-irradiated
Day 7
Day 11
Vehicle control
2 ml/kg CKI
3 ml/kg CKI
Vehicle control
CKI 2ml/kg
CKI 3 ml/kg
Day 7
Day 11

Supplementary figure 4: Administration of CKI have no effect on cell proliferation of irradiated epithelia. Representative IHC images of duodenum (a), jejunum (b), ileum (c) and colon (d) sections stained for Ki-67 and quantitation of Ki-67^{+} cells (top-right). The intestines were collected from irradiated rats on day 7 and 11 post initial irradiation and paraffin-embedded tissues were sectioned for IHC detecting Ki-67. Data are represented as mean±SD.
