# Supplementary material for: Compound Kushen injection reduces severity of radiation-induced gastrointestinal mucositis in rats
Source: Front Oncol. 2022 Aug 11;12:929735. doi: 10.3389/fonc.2022.929735 (PMC9403047; doi:10.3389/fonc.2022.929735)
Supplement: Supplementary file 5 [file Image_5.pdf]

(a) MPO - Duodenum

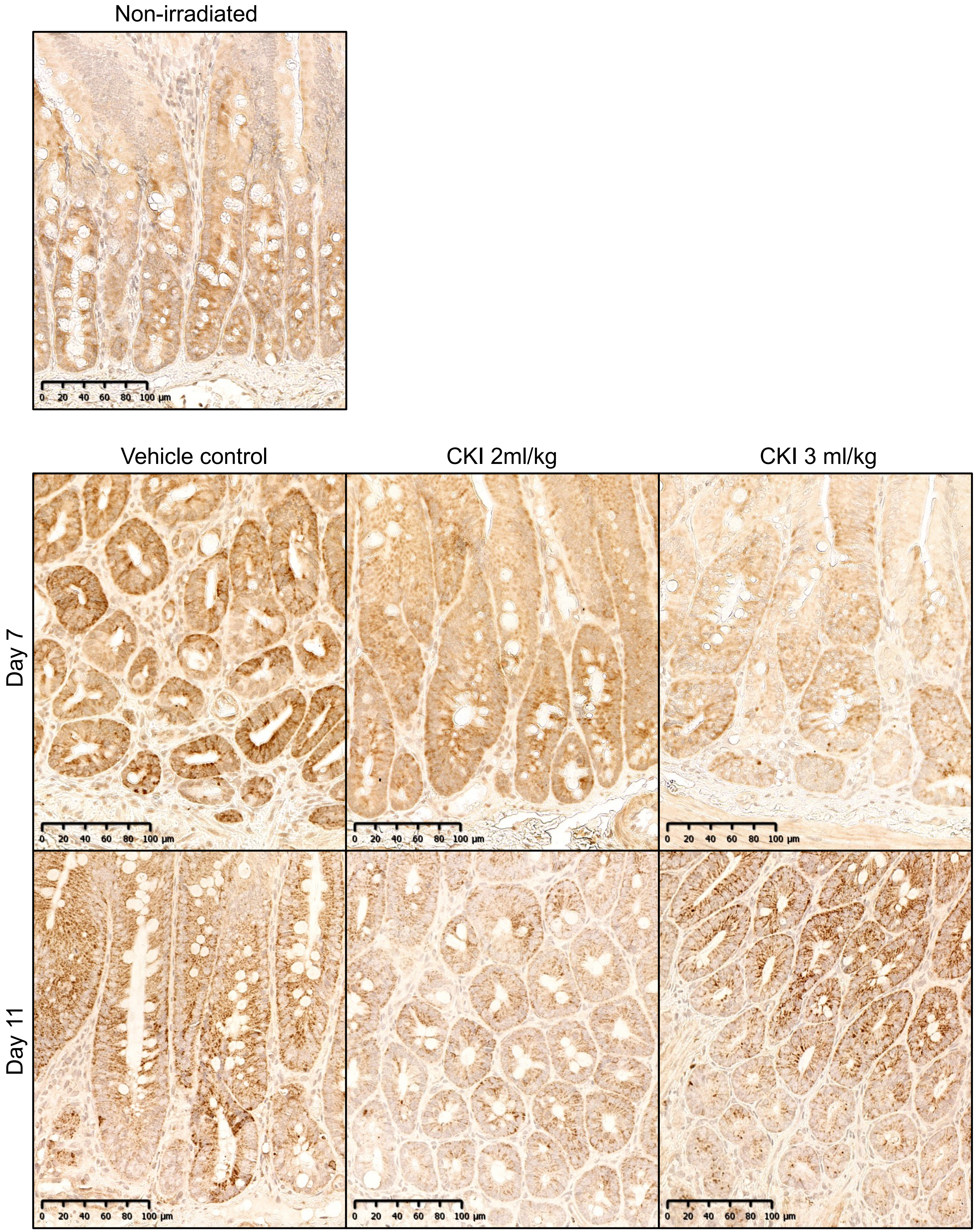

(b) MPO - Jejunum

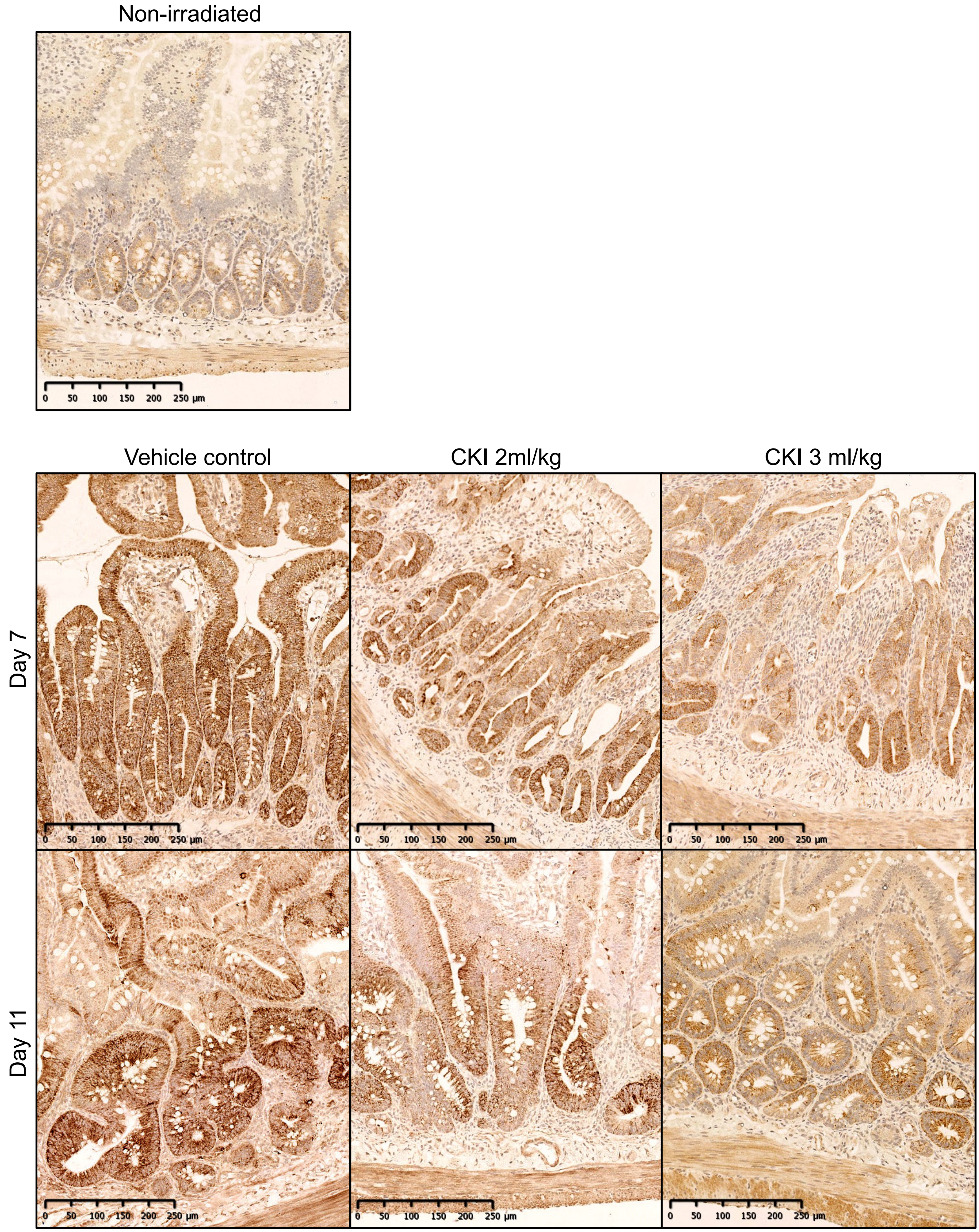

(c) MPO - Ileum

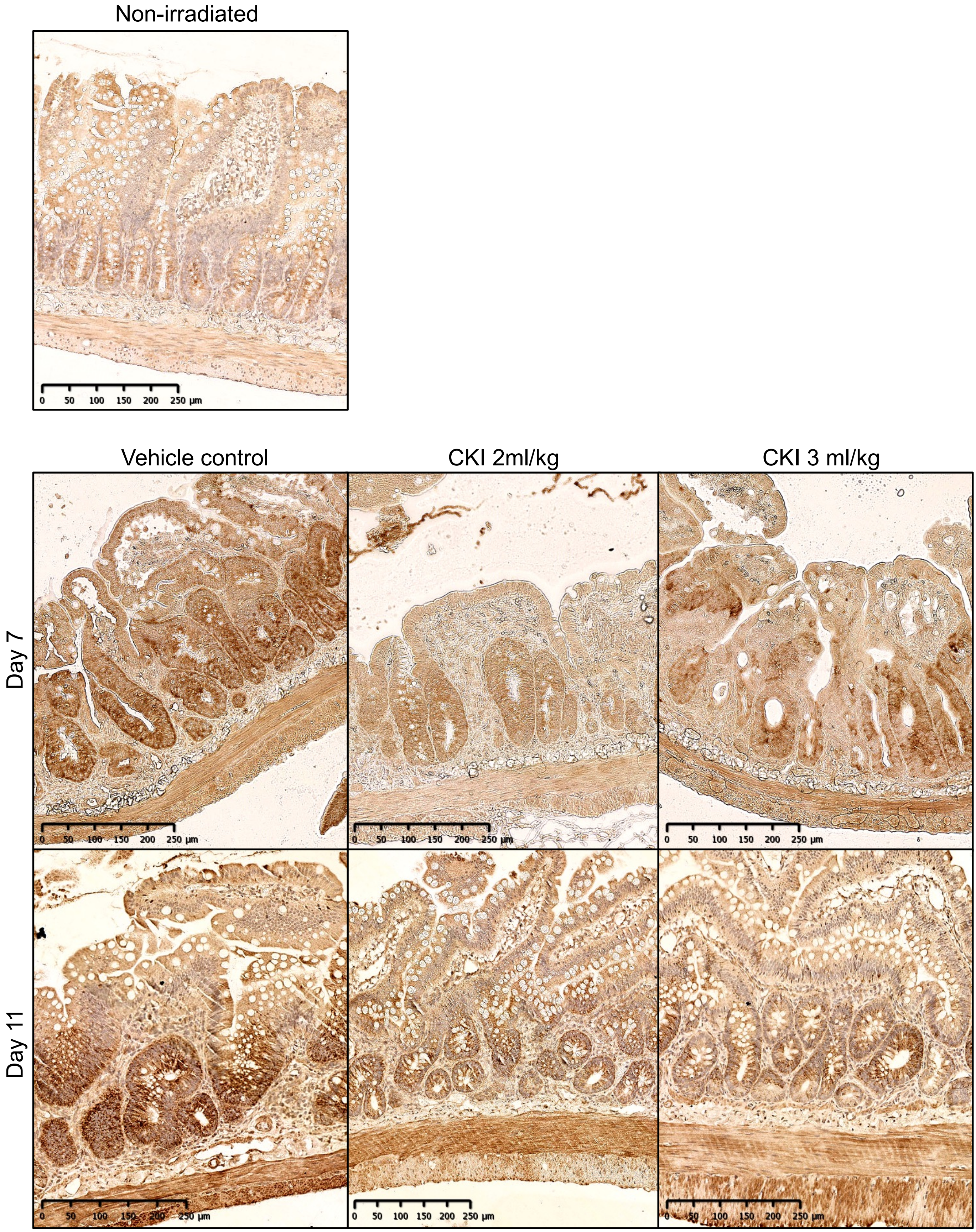
Non-irradiated
Vehicle control
CKI 2ml/kg
CKI 3 ml/kg
Day 7
Day 11
0 50 100 150 200 250 μm

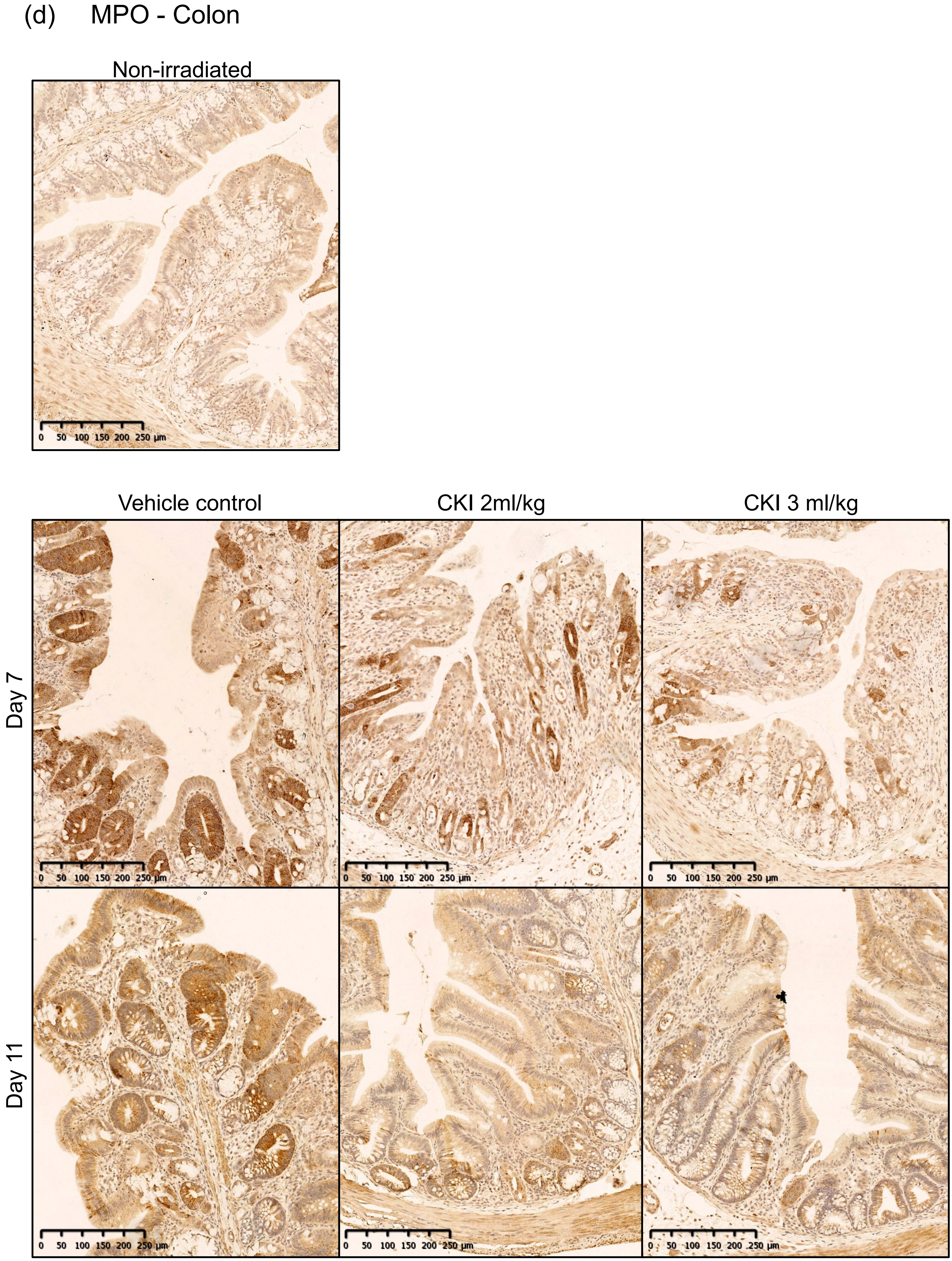

(d) MPO - Colon
Non-irradiated
Vehicle control
CKI 2ml/kg
CKI 3 ml/kg
Day 7
Day 11
0 50 100 150 200 250 µm

(e) IL-1β - Duodenum

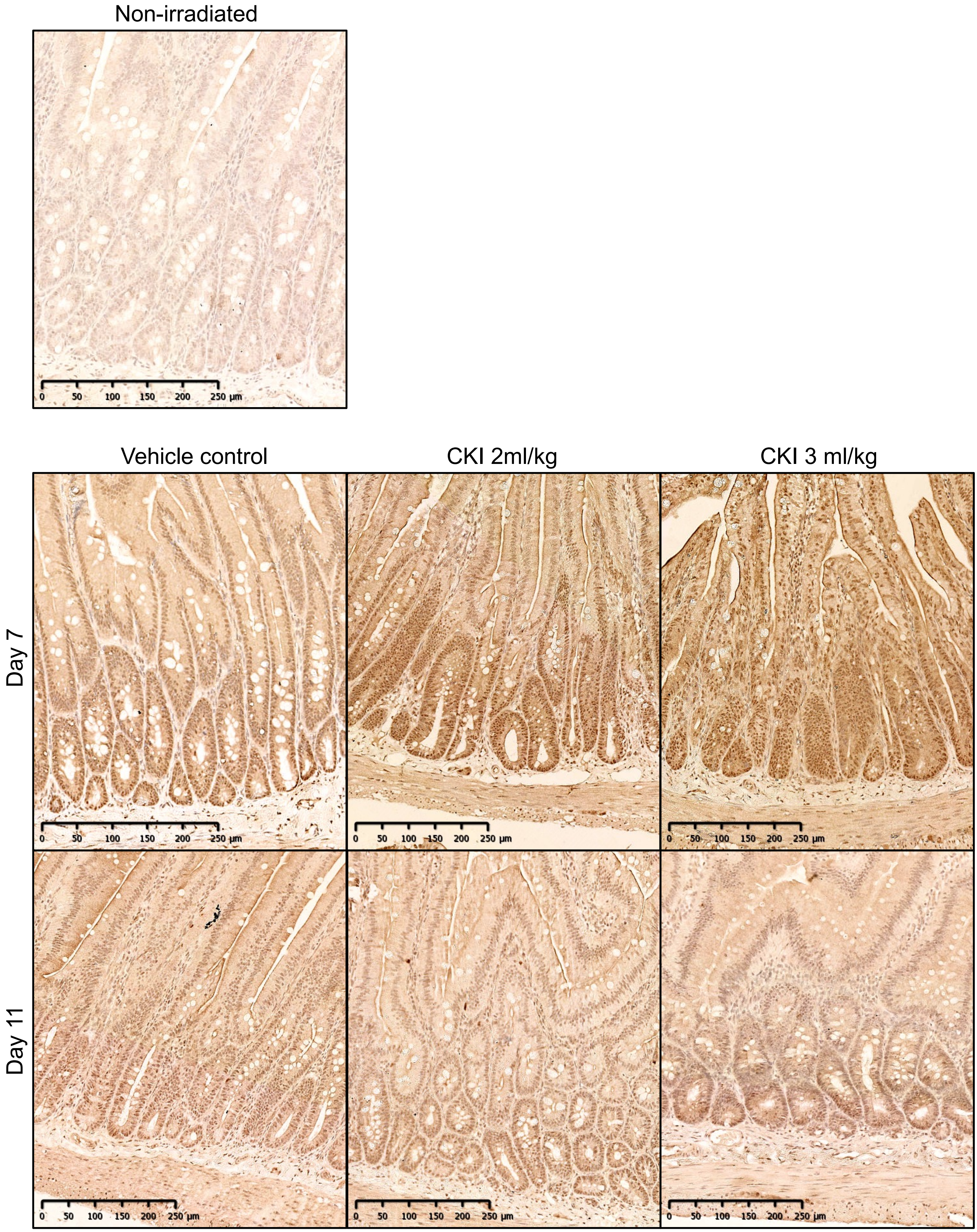

(f) IL-1β - Jejunum

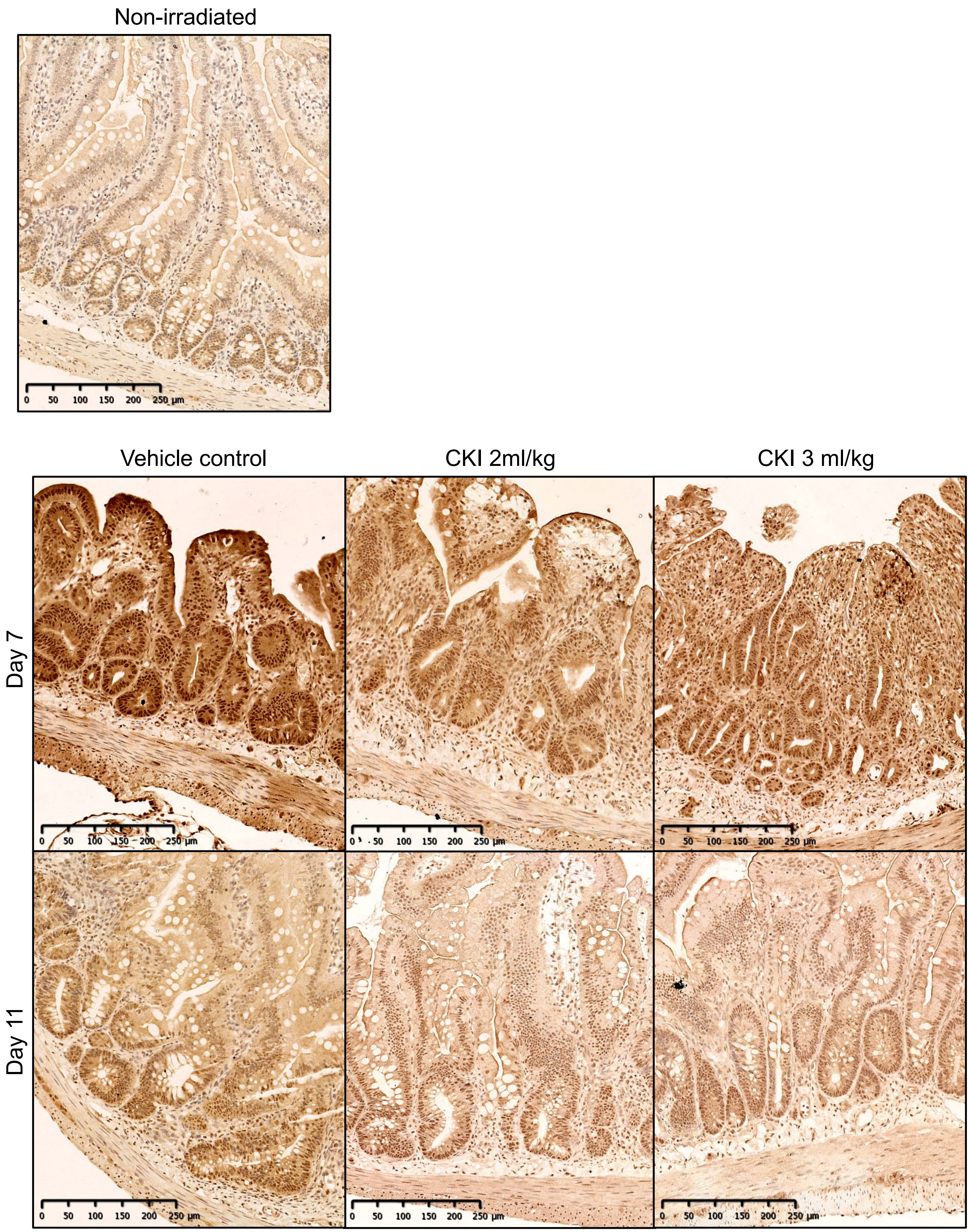

(g) IL-1β - Ileum

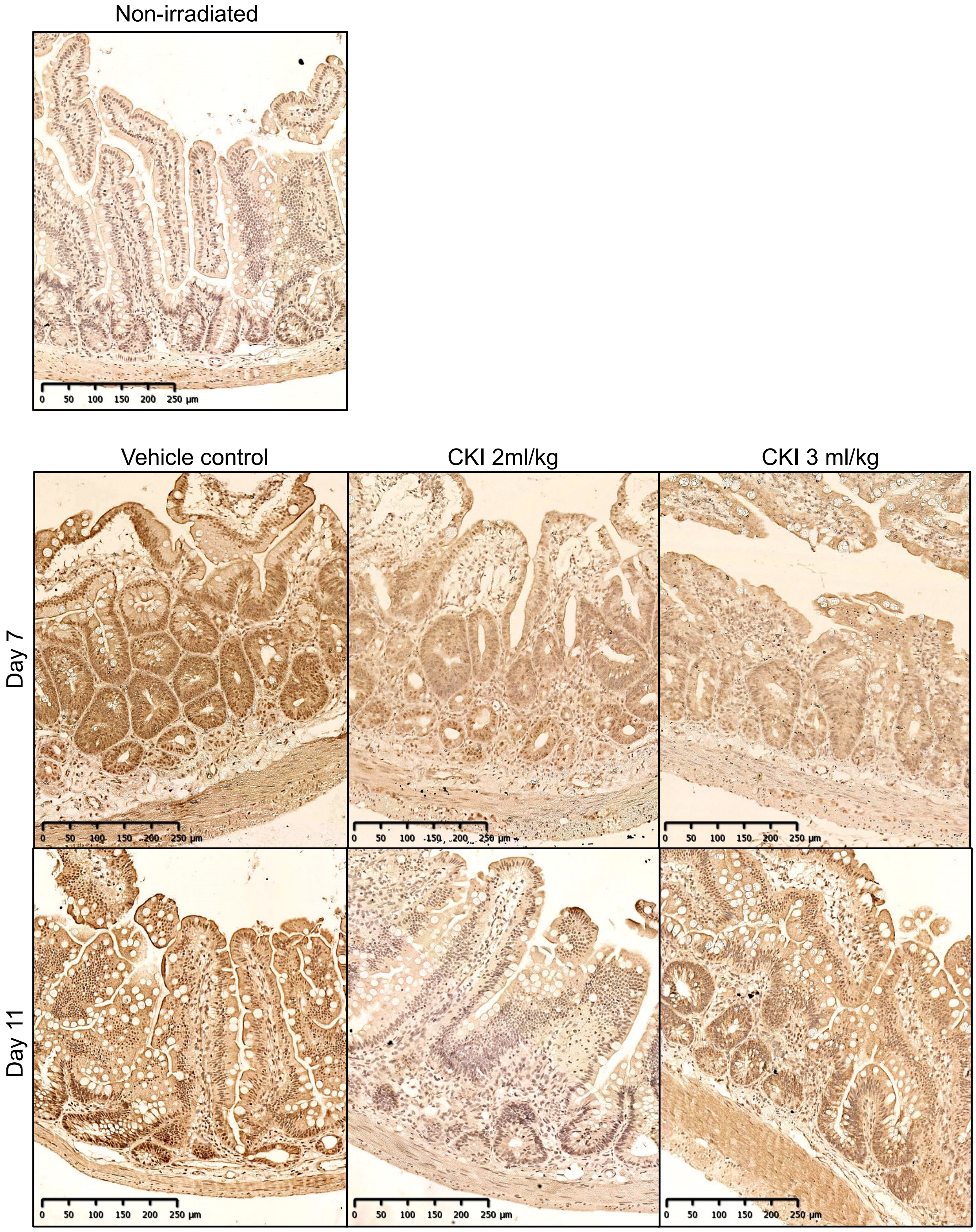

Non-irradiated
Vehicle control
CKI 2ml/kg
CKI 3 ml/kg
Day 7
Day 11
0 50 100 150 200 250 µm
