# Supplementary material for: Compound Kushen injection reduces severity of radiation-induced gastrointestinal mucositis in rats
Source: Front Oncol. 2022 Aug 11;12:929735. doi: 10.3389/fonc.2022.929735 (PMC9403047; doi:10.3389/fonc.2022.929735)
Supplement: Supplementary file 6 [file Image_6.pdf]

(h) IL-1β - Colon

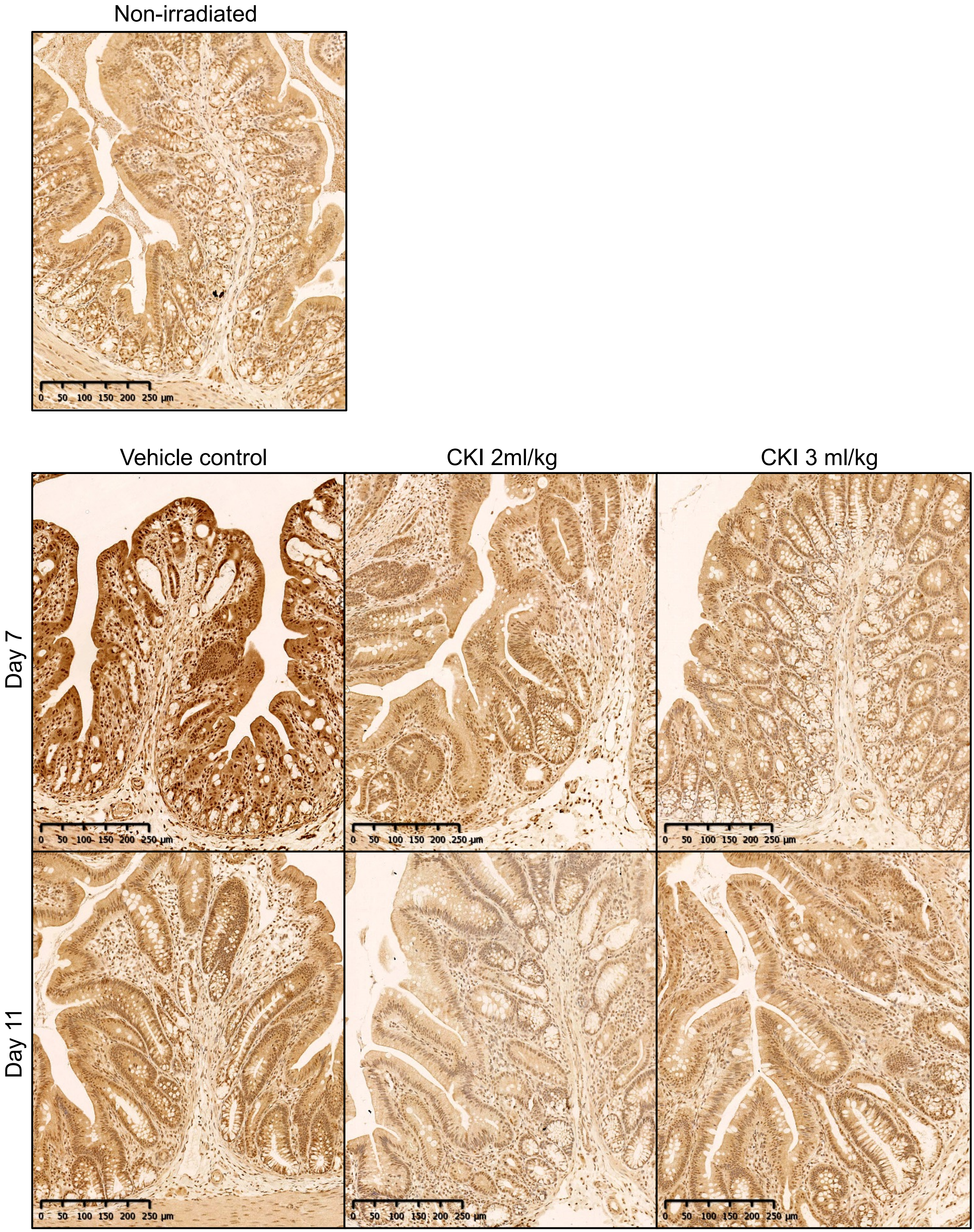

Non-irradiated
Vehicle control
CKI 2ml/kg
CKI 3 ml/kg
Day 7
Day 11
0 50 100 150 200 250 µm

(i) IL-6 - Duodenum

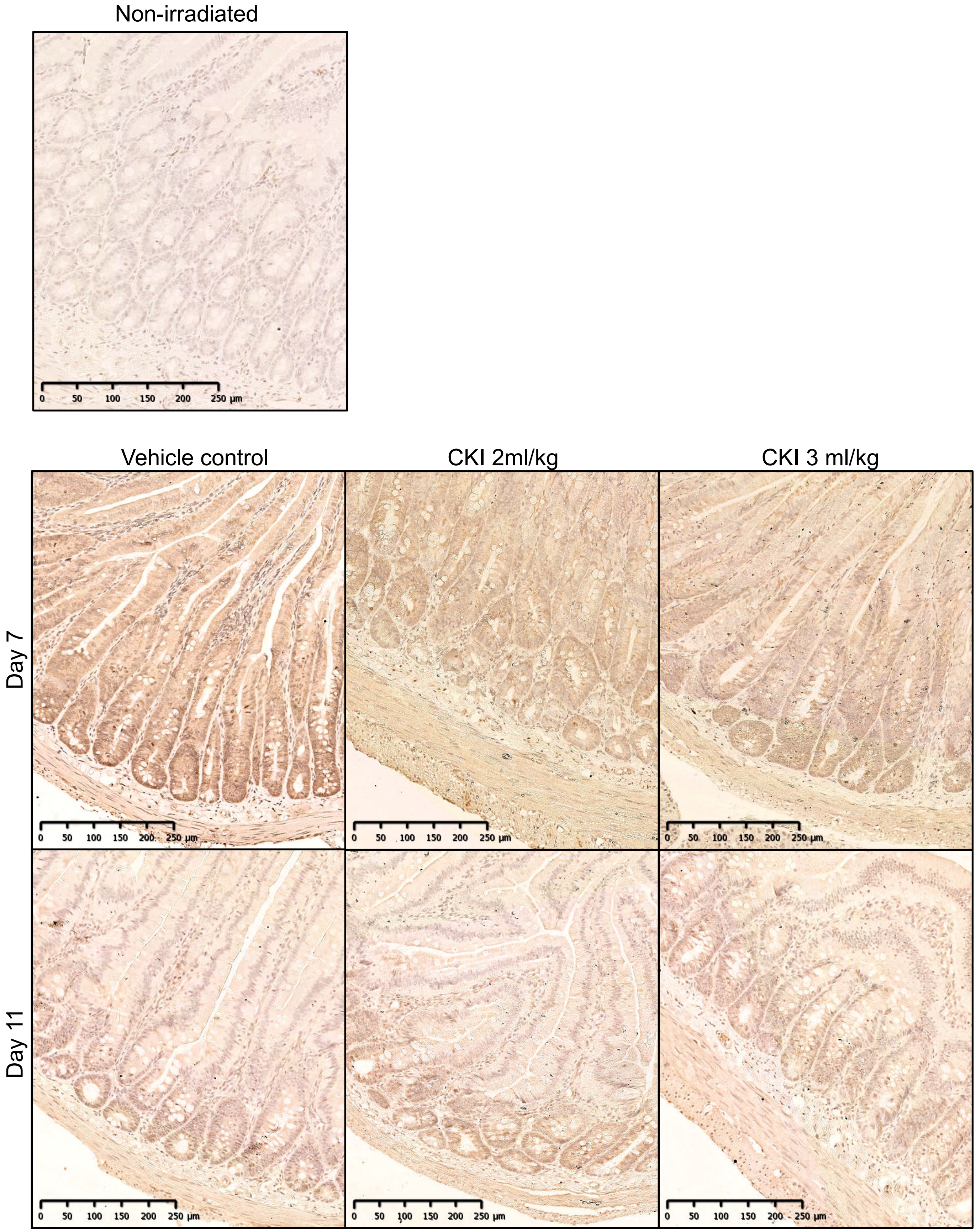

(j) IL-6 - Jejunum

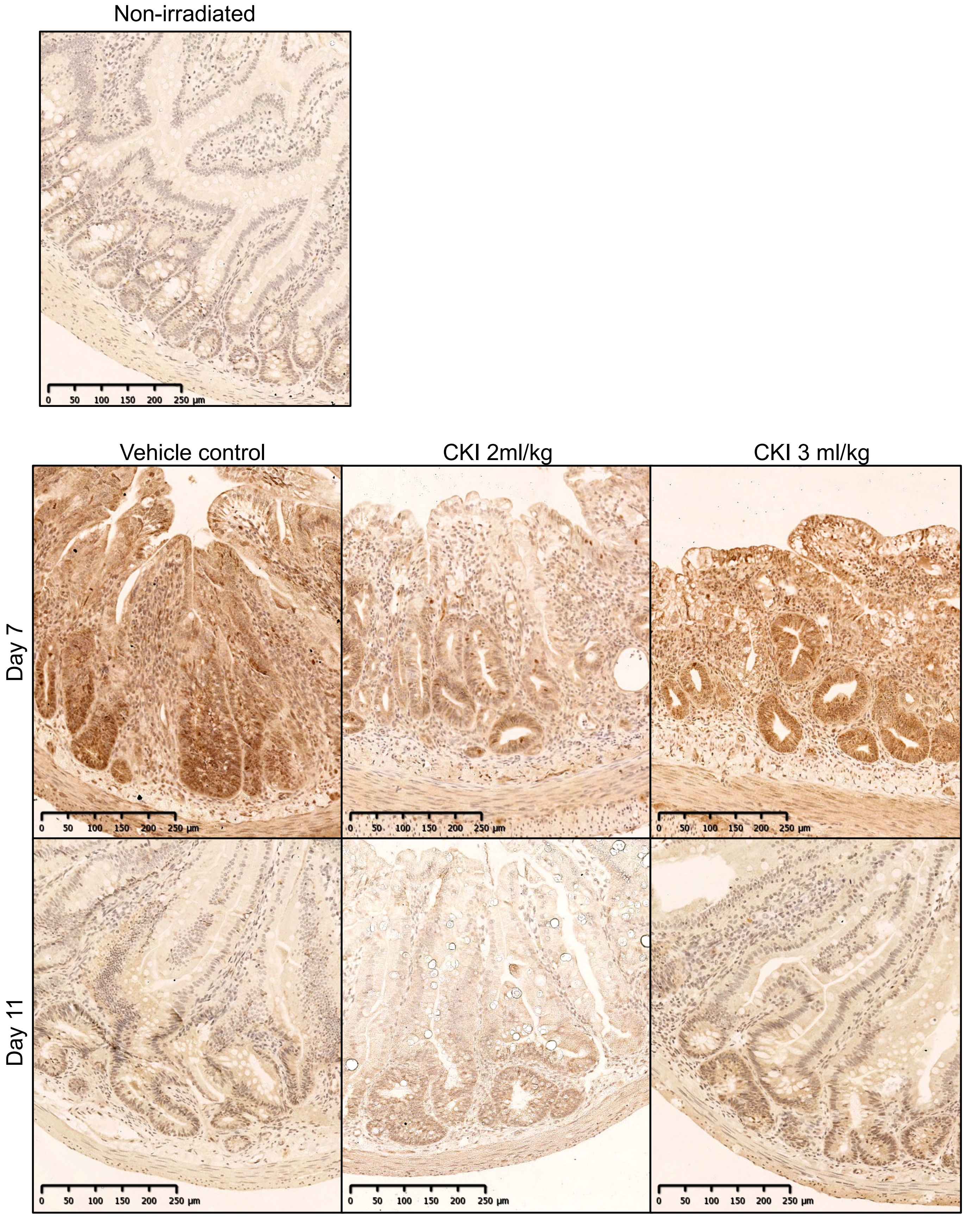

(k) IL-6 - Ileum

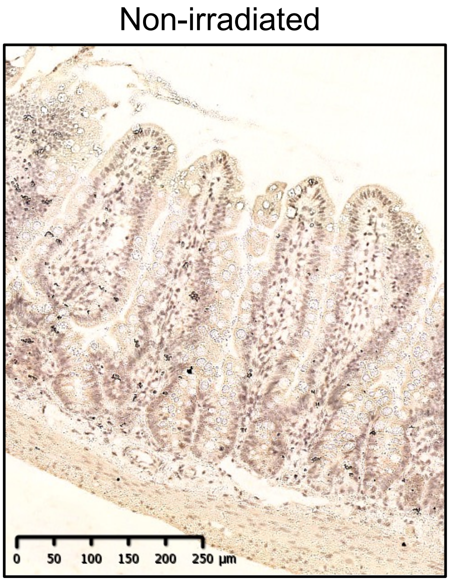

Non-irradiated
0 50 100 150 200 250 µm

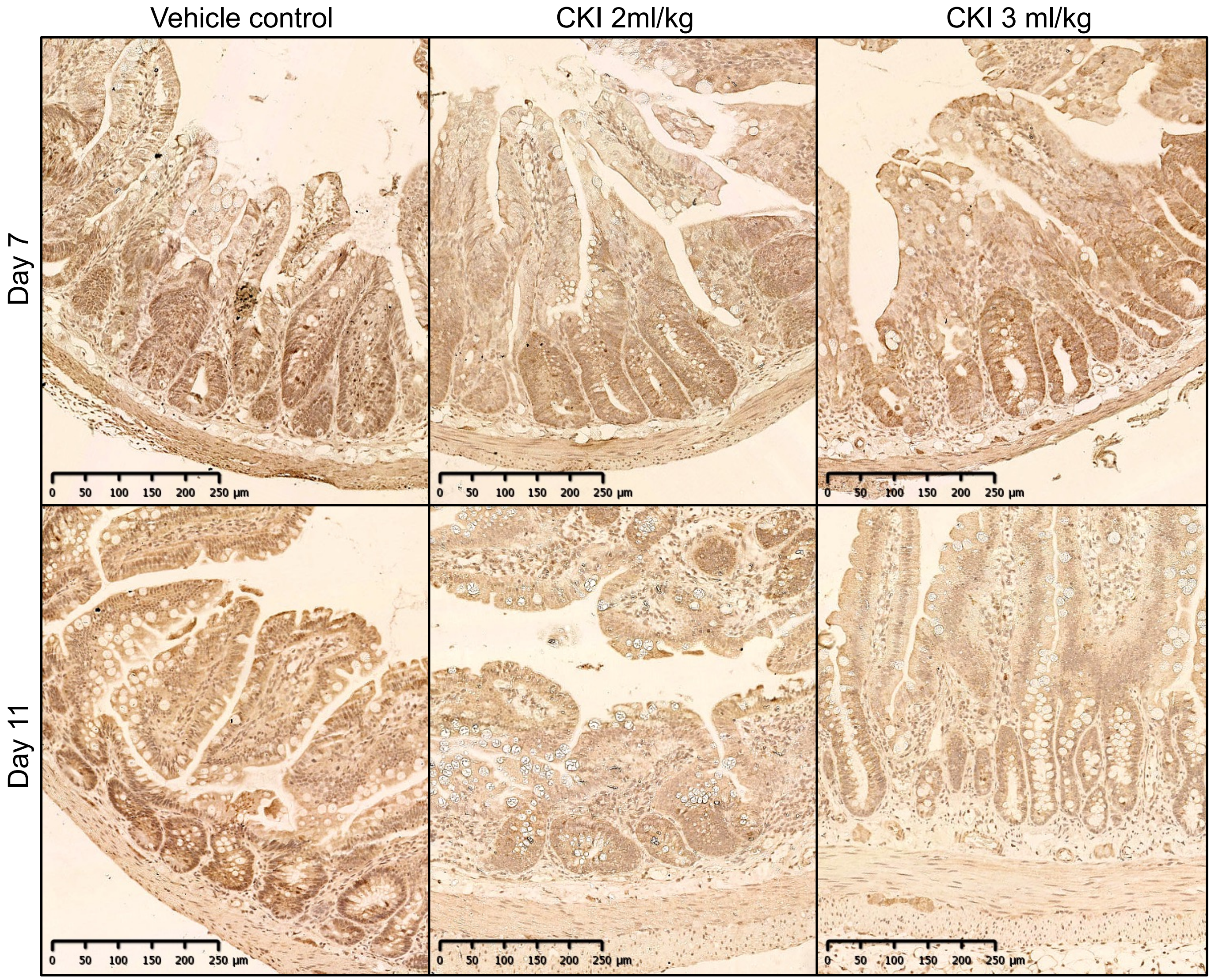

Vehicle control
CKI 2ml/kg
CKI 3 ml/kg
Day 7
Day 11
0 50 100 150 200 250 µm

(I) IL-6 - Colon

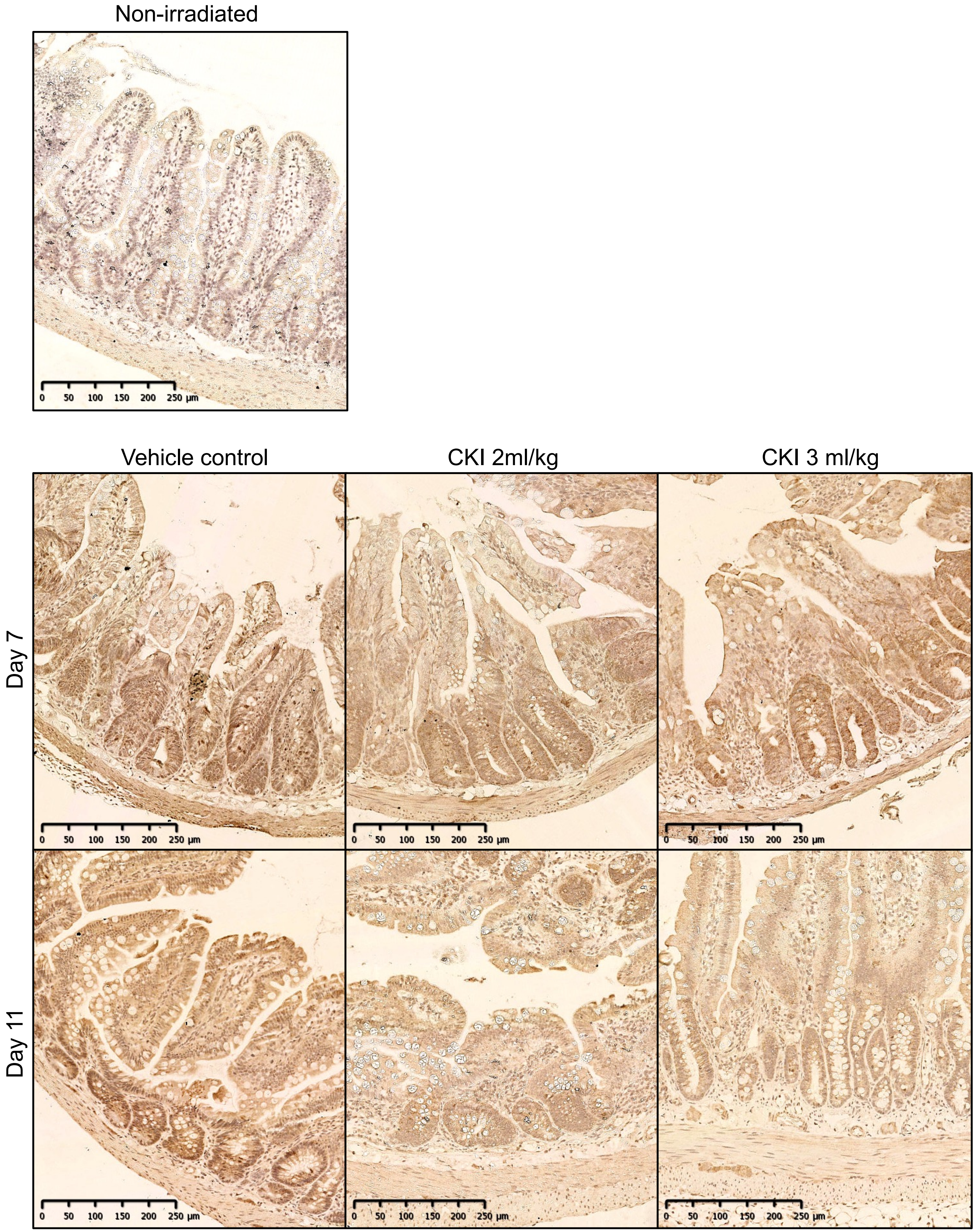

Non-irradiated
Vehicle control
CKI 2ml/kg
CKI 3 ml/kg
Day 7
Day 11
0 50 100 150 200 250 µm

Supplementary figure 5: Administration of CKI reduces radiation induced mucosal inflammation. Representative IHC images of small and large intestines stained with MOP (a-d), IL-1β (e-h), IL-6 (i-l). The intestines were collected from irradiated rats on day 7 and 11 post initial irradiation and paraffin-embedded tissues were sectioned for IHC detecting inflammatory factors indicated.
